# *Klebsiella pneumoniae* LPS drives stromal-mediated repression of p53 and colorectal cancer chemoresistance

**DOI:** 10.1038/s41419-026-08756-4

**Published:** 2026-04-20

**Authors:** Konstantinos Fragkoulis, Barbara Łasut-Szyszka, Ákos Végvári, Diyoly Ayona, Amir Ata Saei, Marie-Stéphanie Aschtgen, Sylvain Peuget

**Affiliations:** 1 Department of Microbiology, Tumor and Cell Biology, Karolinska Institutet, Stockholm, Sweden; 2 Center for Translational Research and Molecular Biology of Cancer, Maria Skłodowska-Curie National Research Institute of Oncology, Gliwice, Poland; 3 Department of Medical Biochemistry and Biophysics, Karolinska Institutet, Stockholm, Sweden; 4 Clinical Microbiology, Karolinska University Hospital, Stockholm, Sweden

**Keywords:** Cancer microenvironment, Tumour-suppressor proteins, Colorectal cancer

## Abstract

The gut bacterial microbiota is increasingly recognized as a key modulator of colorectal cancer (CRC) initiation, progression and response to therapy. However, the mechanisms by which bacteria influence the response to anticancer drugs remain poorly understood. Here, we investigate the effects of microbiota-driven signaling on the tumor suppressor p53 and its impact on chemotherapy. We uncover a mechanism by which lipopolysaccharide (LPS) from *Klebsiella pneumoniae* and other Enterobacteria impairs p53 activity and promotes chemoresistance via paracrine signaling from the tumor microenvironment. While direct exposure to LPS did not alter the drug response of CRC cells, conditioned media from LPS-stimulated macrophages or fibroblasts suppressed p53 accumulation and attenuated the response to chemotherapeutic agents. Deep quantitative proteomics further revealed selective inhibition of a subset of p53 targets by inflammation. This same subset negatively correlated with inflammatory signature and immune infiltration in patients and was associated with improved survival following chemotherapy. Mechanistically, our data suggest that macrophage-derived extracellular vesicles contribute to p53 degradation in cancer cells. Overall, our findings reveal a microbiota-driven mechanism of p53 suppression via the microenvironment that contributes to chemoresistance, highlighting the impact of bacteria on tumor cell fate and therapeutic efficacy in CRC.

## Introduction

Colorectal cancer (CRC) is the second leading cause of cancer-related mortality worldwide [1]. Despite the integration of targeted therapies and immune checkpoint inhibitors, chemotherapy remains central to CRC treatments, typically involving fluoropyrimidines (5-FU, capecitabine) alone or in combination with oxaliplatin- and/or irinotecan (FOLFOX, FOLFIRI, FOLFOXIRI) [2]. However, response rates remain limited due to resistance mechanisms. Emerging evidence implicates the gut microbiota in chemoresistance [3–5]. Specific bacteria, such as *Fusobacterium nucleatum* [6–8] and *Lactobacillus iners* [9], have been associated with resistance, while microbiota modulation can enhance antitumor immunity [10, 11]. Yet, how bacteria influence the efficacy of chemotherapy remains poorly understood.

In CRC, intra-tumoral bacteria colonize spatially organized niches, secrete metabolites and pro-inflammatory factors that reshape the immune landscape and alter host gene expression [12]. Within these niches, p53 levels are significantly reduced, suggesting a correlation between bacterial presence and p53 downregulation [13]. p53 is a key tumor suppressor and regulator of DNA damage response, cell cycle and apoptosis which plays a critical role in response to chemotherapies [14]. p53 inactivation, either by mutation (50-70% of CRC) or by other mechanisms, confers a survival advantage to cancer cells under genotoxic stress [15, 16], although its role in therapy response seems context-dependent [17]. Gut-resident bacteria can alter p53 activity and tumor suppression [18–21], suggesting that bacterial signaling could impact tumor chemoresistance through p53.

CRC patients exhibit gut bacterial dysbiosis, characterized by an overrepresentation of Enterobacteriaceae associated with increased circulating lipopolysaccharide (LPS) [22, 23]. Previously, we showed that LPS from *Klebsiella pneumoniae*, a gut bacteria associated with CRC [24], suppresses p53 expression in fibroblasts and macrophages through TLR4-NF-κB signaling [25]. Here, we investigate the role of *K. pneumoniae* LPS on p53 activity in CRC cells and its impact on chemotherapy. We demonstrate that *K. pneumoniae* LPS does not act directly on CRC cells but rather triggers a paracrine inflammatory circuit mediated by extracellular vesicles (EVs) from activated stromal and immune cells. The associated inflammatory signaling inhibits p53 protein stability and compromises 5-FU-, oxaliplatin- and irinotecan-induced cytotoxicity in vitro. Our analysis of CRC patient transcriptomic data further reveals an inverse correlation between inflammatory gene signatures and p53 pathway activity. These findings emphasize a previously underappreciated role for microbiota-driven inflammation in shaping tumor response to chemotherapy and highlight the tumor microenvironment (TME) as potential therapeutic target to overcome resistance.

## Results

### LPS from Enterobacteria confers chemoprotection to cancer cells via paracrine signals from stromal cells

**Fig. 1:**
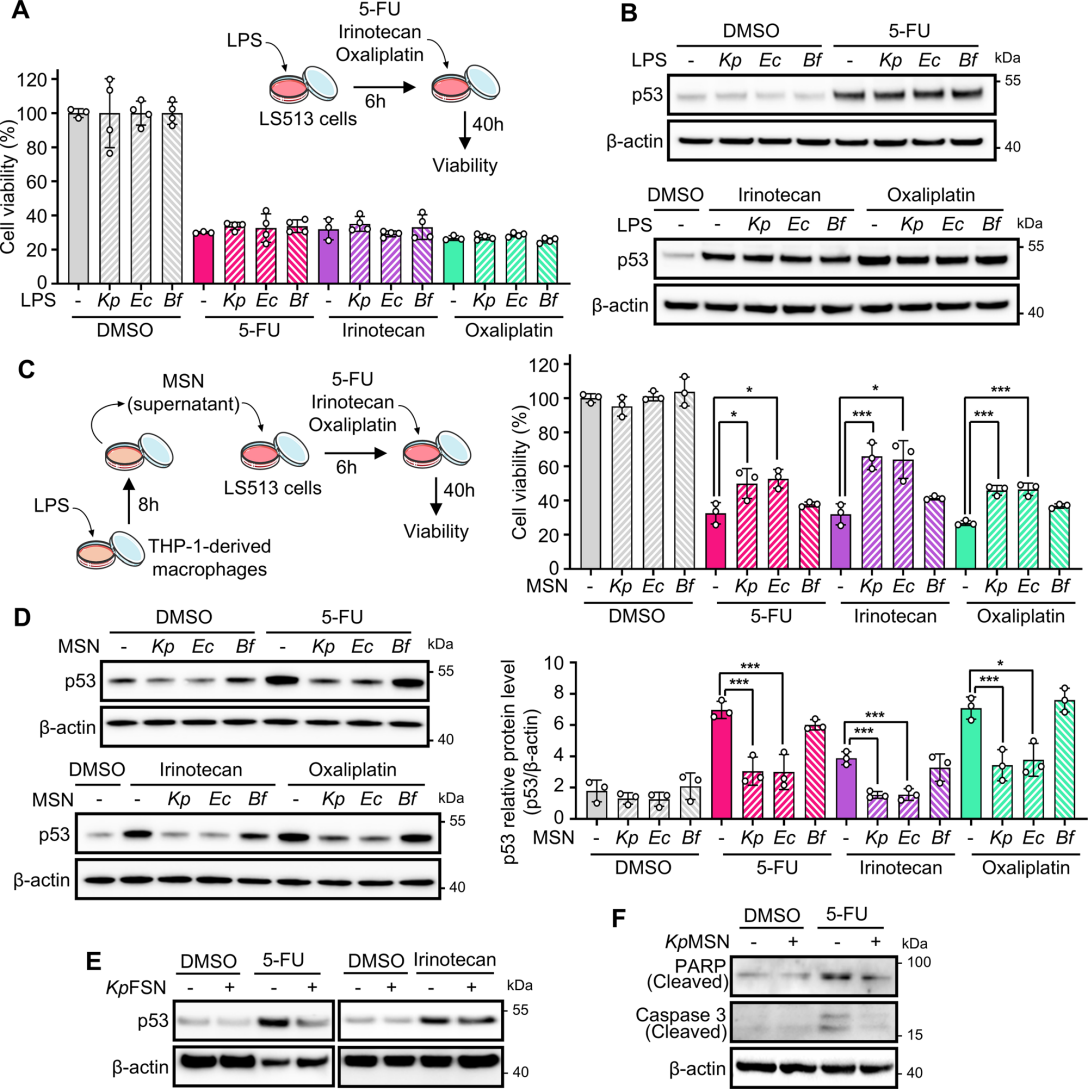
Bacterial LPS modulates chemotherapy response in colorectal cancer cells through paracrine signaling from stromal cells. **A** LS513 colorectal cancer cells treated with LPS (100 ng/mL) from *Klebsiella pneumoniae* (*Kp*), *Escherichia coli* (*Ec*), or *Bacteroides fragilis* (*Bf*) for 6 h, followed by 5-FU (1 μM), irinotecan (5 μM), or oxaliplatin (1 μM) for an additional 40 h. Cell viability was measured by resazurin assay. No significant changes were observed upon LPS treatment. **B** Western blot analysis of p53 protein levels in LS513 cells exposed to chemotherapeutic drugs following 6 h LPS pretreatment. **C** LS513 cells were pre-incubated for 6 h with macrophage supernatants (MSN) derived from THP-1 cells stimulated for 8 h with LPS from *Kp*, *Ec*, or *Bf*, then treated with chemotherapy agents. Cell viability was assessed by resazurin. **D** Western blot of LS513 cells pre-treated with MSN and then exposed to chemotherapeutic agents. Right panels show the densitometry of the bands from three independent replicates. **E** Western blot of LS513 cells treated with *Kp* LPS-stimulated BJ hTERT fibroblasts (*Kp*FSN) prior to 5-FU or irinotecan. **F** Apoptosis assessed by Western blot for PARP and Caspase-3 cleavage in LS513 cells treated with 5-FU and *Kp*MSN. Data are representative of at least three independent experiments. *p < 0.05; ***p < 0.01.

Given our previous finding that LPS represses p53 via the TLR4–NF-κB axis in fibroblasts and macrophages [25], we investigated whether a similar mechanism operates in CRC cells. We used LS513 cells, expressing wild-type (WT) p53 and high TLR4 levels [26], as main model (Supplementary Fig. S1A–C). LS513 cells were treated with LPS purified from either TLR4-activating Enterobacteria (*Klebsiella pneumoniae* and *Escherichia coli*) or from non-toxigenic *Bacteroides fragilis*, whose penta-acylated lipid A structure does not activate TLR4 [27]. Cell viability under 5-FU, irinotecan, or oxaliplatin was assessed by resazurin assay. Unexpectedly, LPS had no effect on drug-induced cytotoxicity (Fig. 1A and Supplementary Fig. S2A) or p53 protein levels (Fig. 1B), suggesting that direct LPS signaling is insufficient to modulate p53 or chemotherapy response in CRC cells. In line with this result, we observed an impaired NF-κB response to *K. pneumoniae* LPS in CRC cell lines irrespective of TLR4 level (Supplementary Fig. S1C).

We hypothesized that the LPS-mediated inhibition of p53 in stromal cells might act via paracrine mechanisms. To test this, we stimulated THP-1-derived macrophages with LPS, and exposed LS513 cells to the conditioned macrophage supernatant (MSN) prior to treatment with chemotherapeutic agents. We found that *K. pneumoniae* or *E. coli*-stimulated MSN but not *B. fragilis* MSN provided significant chemoprotection to LS513 cells (Fig. 1C and Supplementary Fig. S2A), correlating with an impaired stabilization of p53 upon drug treatment (Fig. 1D). This effect was reproduced in p53 WT SK-CO-1 and RKO cells but not in p53 mutant SW480 cells, (Supplementary Fig. S2B–D), and with conditioned supernatant from *K. pneumoniae* LPS-stimulated fibroblasts (Fig. 1E). Moreover, *K. pneumoniae*-stimulated MSN (*Kp*MSN) reduced 5-FU-induced apoptosis, as measured by PARP and Caspase-3 cleavage (Fig. 1F).

We then assessed the importance of the LPS–TLR4 pathway in THP-1 cells for p53 downregulation in cancer cells. Similar to purified LPS, crude supernatant from *K. pneumoniae* cultures (*Kp*SN) had no direct effect on p53 levels in LS513 cells; however, MSN generated from *Kp*SN-stimulated THP-1 cells induced p53 downregulation. In contrast, MSN generated from *K. pneumoniae* Δ*lpxM* strain, which lacks functional LPS [25], failed to suppress p53 (Supplementary Fig. S3B–D). Consistently, pharmacological inhibition of TLR4 in THP-1 cells using TAK-242 abrogated MSN-mediated p53 downregulation (Supplementary Fig. S3E–G), indicating that LPS is the primary bacterial driver of this paracrine effect in our model.

Altogether, our results support a model in which bacteria attenuate p53 response in cancer cells through the TME.

### LPS-stimulated macrophage signaling impairs cell cycle and p53 activity in CRC cells

**Fig. 2:**
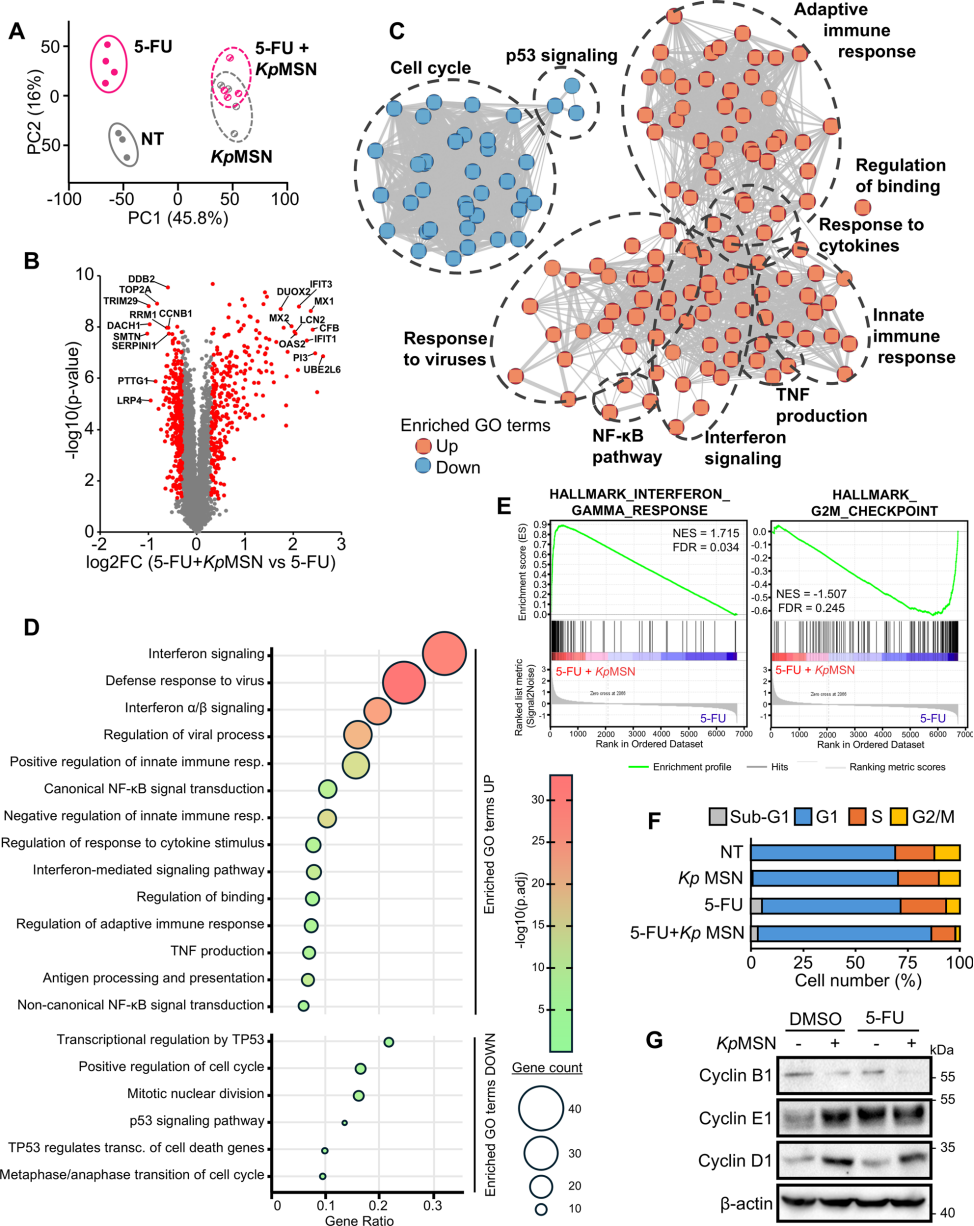
LPS-stimulated macrophage supernatant impairs p53 activity and induces cell cycle arrest in colorectal cancer cells. **A** PCA of proteomic data from LC-MS/MS performed on LS513 cells treated with vehicle (NT), 5-FU, *Kp*MSN, or combination. **B** Volcano plot comparing 5-FU + *Kp*MSN versus 5-FU conditions. Top differentially abundant proteins are indicated. **C** Network visualization of the significantly up- and downregulated pathways in Gene Ontology (GO) analysis of differentially abundant proteins upon 5-FU and 5-FU+*Kp*MSN conditions. **D** Top up- and downregulated GO pathways. **E** Top pathways enriched in GSEA analysis. Cell cycle distribution of LS513 cells treated with vehicle (NT), 5-FU, *Kp*MSN, or combination, assessed by flow cytometry (**F**) or by Western blot for major cyclins (**G**).

To investigate how macrophage-derived factors impact chemotherapy response in CRC cells, we performed deep quantitative proteomics using tandem mass tag (TMT)-based mass spectrometry (MS) on LS513 cells treated with 5-FU, *Kp*MSN, or their combination (Fig. 2 and Supplementary Fig. S4A–E; Supplementary Table S1). Principal component analysis (PCA) revealed distinct clustering for untreated, 5-FU- and *Kp*MSN-treated samples, whereas the 5-FU+*Kp*MSN group partially overlap with the *Kp*MSN-alone condition (Fig. 2A), indicating that *Kp*MSN shapes cancer cell response. Gene Ontology (GO) pathway analysis of the differentially abundant proteins upon 5-FU and 5FU+*Kp*MSN conditions (Fig. 2B–D) showed an upregulation of the immune response, including interferon signaling and NF-κB pathway, and a repression of p53 signaling, including cell cycle progression and mitotic cell division. Gene Set Enrichment Analysis (GSEA) using the Molecular Signatures Database (MSigDB) hallmark gene sets [28] confirmed the enrichment of interferon pathway and downregulation of G2/M checkpoints (Fig. 2E), suggesting that *Kp*MSN induce a cell cycle arrest in LS513 protecting them from chemotherapeutic drug-induced apoptosis. Cell cycle analysis by flow cytometry and by immunoblotting confirmed G1/S arrest in LS513 cells upon 5-FU+*Kp*MSN treatment (Fig. 2F, G and Supplementary Fig. S4F).

**Fig. 3:**
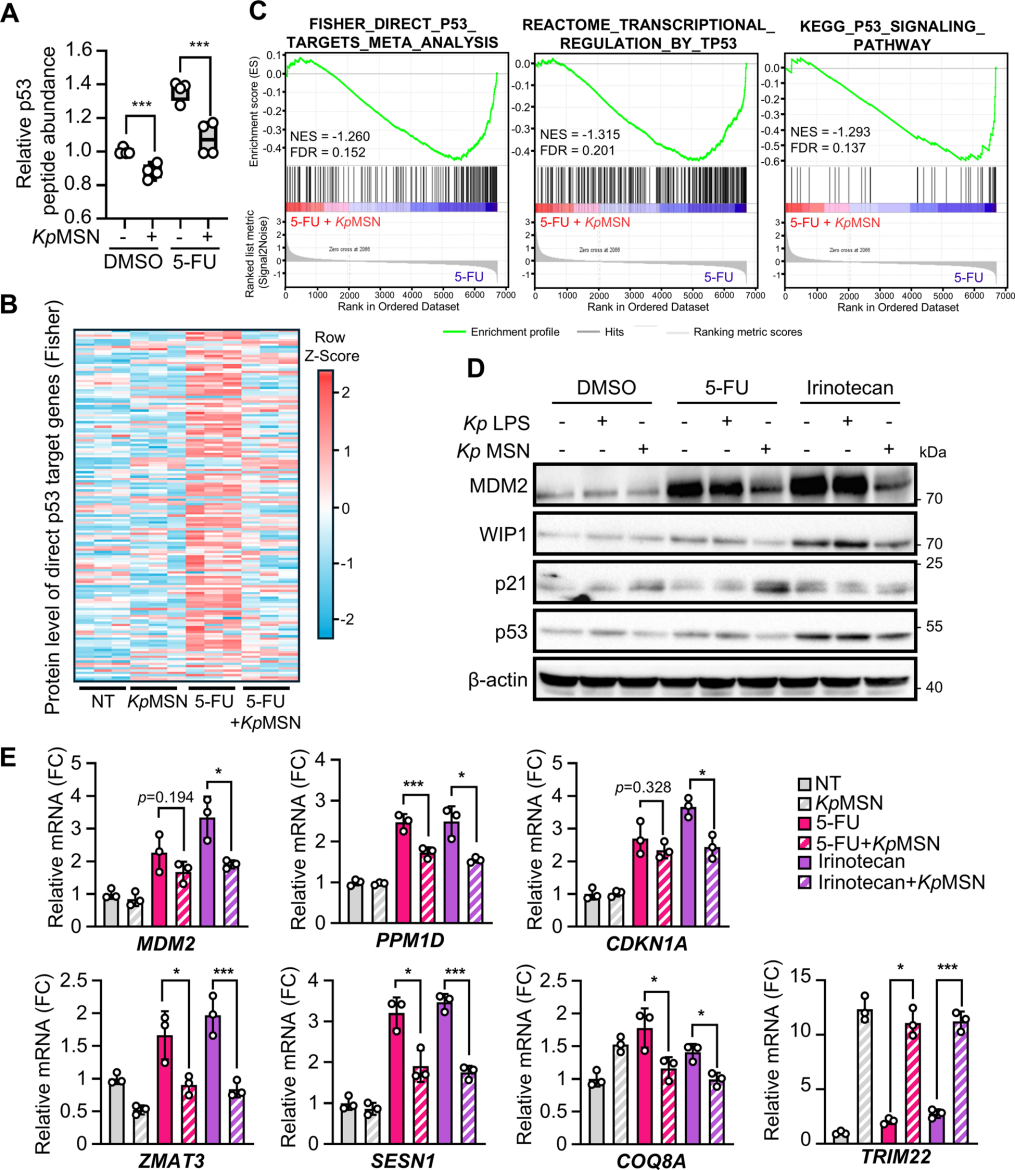
LPS-stimulated macrophage supernatant impairs p53 activity in colorectal cancer cells. **A** p53 protein abundance detected by LC-MS/MS in LS513 cells following exposure to *Kp*MSN and 5-FU. **B** Abundance of 154 direct p53 targets detected by LC-MS/MS across treatment conditions. **C** GSEA for p53 direct targets and p53 signaling pathway comparing 5-FU+*Kp*MSN versus 5-FU treated LS513. **D** Level of direct p53 targets MDM2, WIP1 and p21 assessed by Western blot in LS513 cells treated with 5-FU or irinotecan after exposure to *Kp* LPS or *Kp*MSN. **E** qPCR analysis of canonical p53 target genes (*MDM2, PPM1D, ZMAT3, SESN1, COQ8A, CDKN1A*) in LS513 cells. *p < 0.05; ***p < 0.01.

We next investigated how *Kp*MSN influences p53 response in LS513 cells. A significant decrease in p53 protein following *Kp*MSN exposure was confirmed in our proteomics data (Fig. 3A). To assess the functional consequences on p53 activity, we examined the protein levels of direct p53 target genes from the Fisher gene set [29]. Comparison of the 154 direct p53 targets detected at our proteomics depth revealed a striking impairment of p53 response to 5-FU upon treatment with *Kp*MSN (Fig. 3B). GSEA confirmed a significant depletion of direct p53 targets upon *Kp*MSN and 5-FU combination treatment compared to 5-FU alone. These findings were cross-validated using gene sets for downstream p53 signaling from Reactome and KEGG databases (Fig. 3C). We then confirmed these results by Western blot, showing impaired induction of p53 targets MDM2 and WIP1 after 5-FU and irinotecan treatment in the presence of *Kp*MSN, but not directly upon exposure to *K. pneumoniae* LPS (Fig. 3D and Supplementary Fig. S5A, B). Consistent with the G1/S cell cycle arrest observed in *Kp*MSN-treated cells, the canonical p53 target p21 was robustly induced by *Kp*MSN independently of p53 activation.

We then further assessed p53 transcriptional activity by qPCR and confirmed that 5-FU and irinotecan-induced upregulation of p53 targets, including *MDM2*, *PPM1D* (WIP1), *ZMAT3*, *SESN1* and *COQ8A*, was significantly impaired by *Kp*MSN (Fig. 3E). Similar results were observed for *CDKN1A* (p21), despite its upregulation by *Kp*MSN at protein level, suggesting transcriptional regulation by both p53-dependent and p53-independent mechanisms. This was further supported by analysis at an early time point following *Kp*MSN treatment (Supplementary Fig. S5C). In contrast, *TRIM22*, a p53 target co-regulated by interferon signaling [30, 31], was robustly induced by *Kp*MSN regardless of chemotherapeutic treatment (Fig. 3E), indicating that bacterial-elicited inflammatory response controls a subset of the p53 transcriptome.

### Downregulation of p53 activity correlates with inflammation and immune infiltration in CRC patients

**Fig. 4:**
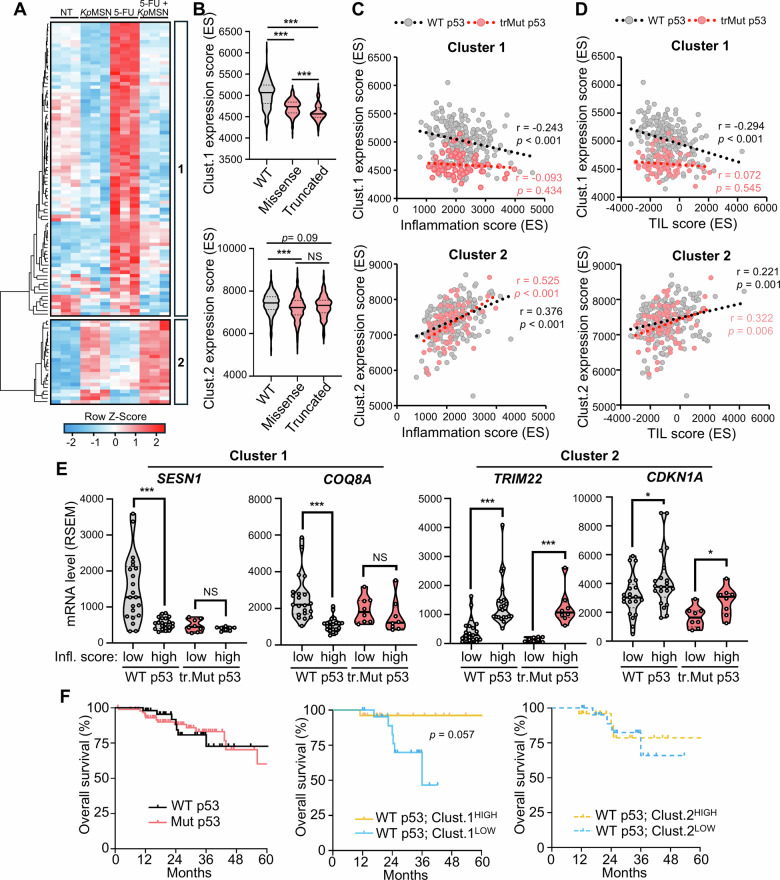
p53 activity is suppressed in colorectal tumors with high inflammation. **A** Unsupervised hierarchical clustering of differentially abundant direct p53 targets in our LS513 LC-MS/MS data. **B** Expression signature of Cluster 1 and Cluster 2 scored by ssGSEA in colorectal tumors from the TCGA PanCancer Atlas according to p53 mutation status. **C** Correlation of inflammation score with Cluster 1 and Cluster 2 scores in p53 WT and mutant tumors. **D** Similar correlations using tumor-infiltrating lymphocyte (TIL) scores. **E** Comparison of gene expression for selected p53 targets from Cluster 1 (*SESN1* and *COQ8A*) and Cluster 2 (*TRIM22* and *CDKN1A*) in CRC tumors with high versus low inflammation scores in p53 WT and p53 null (truncated mutant) tumors. **F** Survival analysis of chemotherapy-treated CRC patients from the TCGA cohort, stratified by p53 status and by high versus low Cluster 1 and Cluster 2 expression. *p < 0.05; ***p < 0.01; NS: not significant.

We next asked whether p53 activity was modulated by inflammatory signals from the TME in CRC patients. Unsupervised clustering of the differentially abundant p53 targets in our LS513 proteomics data revealed two distinct clusters of p53 target genes (Fig. 4A and Supplementary Table S2): cluster 1 reflects p53 inhibition under inflammatory conditions, while cluster 2 comprises the subset of p53 targets induced in response to inflammatory cues. Cluster 1 genes were strongly induced by 5-FU alone, and this activation was prevented by *Kp*MSN. On the other hand, expression of cluster 2, albeit also responsive to 5-FU, was predominantly driven by *Kp*MSN.

We then explored the expression of each cluster in CRC patients' data from the TCGA. Single-sample GSEA (ssGSEA) was used to score the expression of each cluster in individual tumors. To validate that both clusters were regulated by p53, we compared their expression score in p53 WT, missense mutant or truncated mutant (p53 null) tumors. Cluster 1 was tightly associated with p53 activity (Fig. 4B), lower in mutant p53 compared to WT tumors, and lower in tumors with truncating mutations compared to missense mutations that retain a partial transcriptional activity. Cluster 2 expression was also reduced in p53-mutant tumors, although less pronounced.

To examine the correlation of each cluster with inflammation, we calculated an inflammation score for each tumor using ssGSEA for the hallmark gene set Inflammatory Response from MSigDB (Fig. 4C). In tumors with wild-type p53, cluster 1 expression negatively correlated with inflammation, but not in tumors with truncating p53 mutations, suggesting that inflammation represses cluster 1 in a p53-dependent manner. Cluster 2 expression was positively associated with inflammation in a p53-independent manner, consistent with our in vitro data. Similar results were obtained when comparing each cluster expression to a tumor-infiltrating lymphocyte (TIL) score based on the expression of immune cell-specific genes [32], strongly supporting that the inflammatory signal regulating p53 in the tumor is a paracrine signal from the TME (Fig. 4D and Supplementary Fig. S6). Comparison of gene expression for selected p53 targets from cluster 1 and 2 in tumors with low or high inflammation score was consistent with our in vitro proteomics data (Fig. 4E).

In line with previous studies [33, 34], p53 mutation status alone was not predictive of overall survival in CRC patients treated with 5-FU, oxaliplatin, or irinotecan in mono- or combination therapy (Fig. 4F). However, high cluster 1 expression in WT p53 tumors correlated with survival, while low cluster 1 expression had worse outcomes, even compared with mutant p53 tumors. We did not observe any correlation between cluster 2 and survival. Although not statistically significant (*p* = 0.057) due to limited size of the TCGA cohort, the trend suggests that inflammation-mediated suppression of p53 contributes to poor clinical response in WT tumors and warrants further investigation in larger cohorts and in vivo validation. Overall, our data revealed a gene signature reflecting p53 activity in inflammation conditions and correlates with patient response to chemotherapy.

### LPS-stimulated macrophage supernatant impairs p53 activity through protein destabilization

**Fig. 5:**
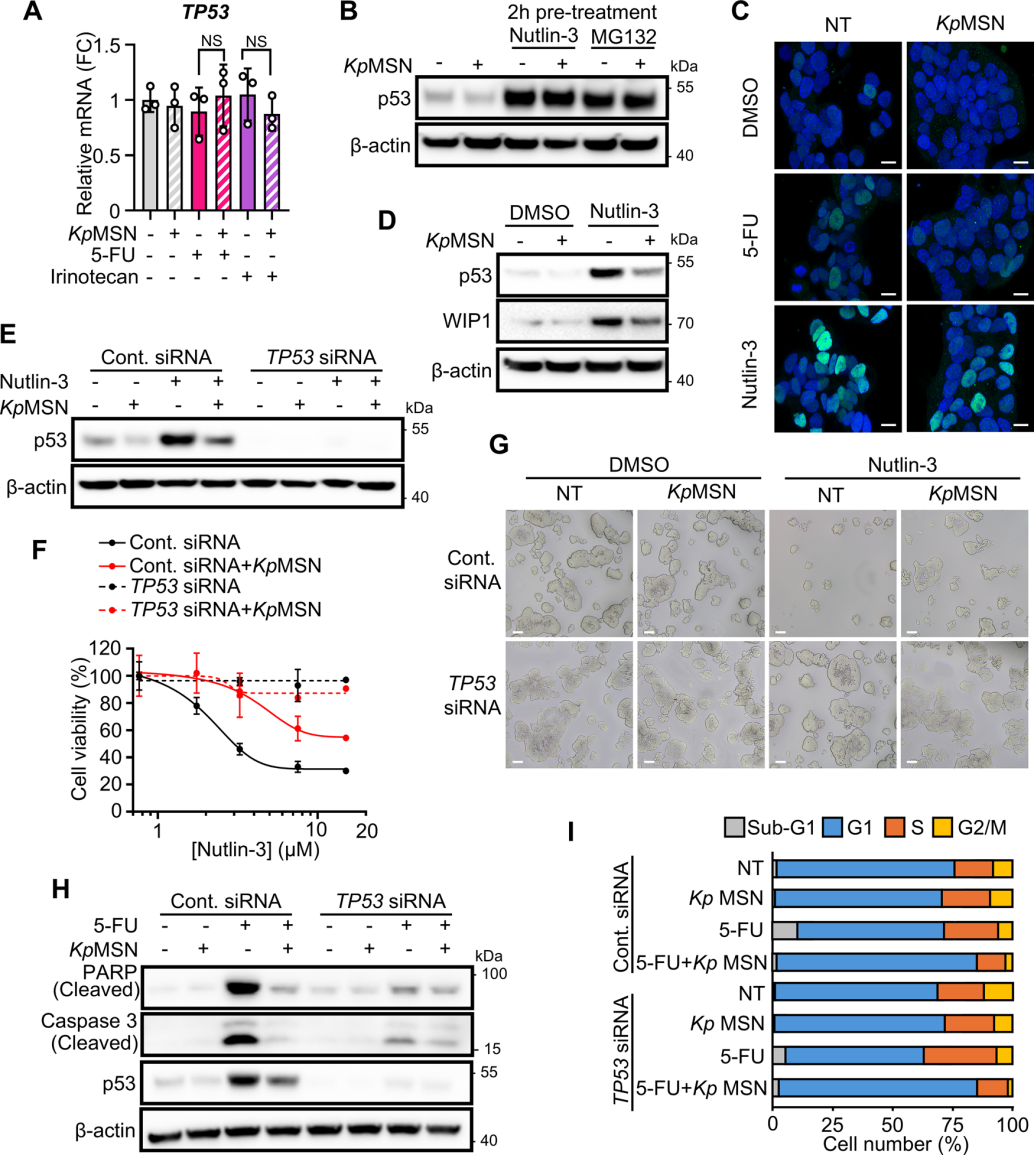
LPS-stimulated macrophage signaling impairs p53 stability and p53-dependent apoptosis. **A** Relative expression of *TP53* in LS513 cells treated with *Kp*MSN followed by 5-FU or Irinotecan, measured by RT-qPCR. **B** Western Blot of LS513 cells pretreated with Nutlin-3 or MG132, followed by *Kp*MSN treatment. **C** p53 immunofluorescence of LS513 cells exposed to *Kp*MSN and then treated with 5-FU or Nutlin-3. Scale bar = 10 μm. **D** Western Blot of LS513 exposed to *Kp*MSN for 6 h prior to Nutlin-3 treatment. **E** Validation of p53 depletion in LS513 cells transfected with *TP53* siRNA or control (Cont. siRNA), assessed by Western blot. **F** Cell viability of LS513 cells measured by resazurin assay upon Nutlin-3 treatment. p53-dependency is assessed using *TP53* siRNA. **G** Representative images of LS513 cells transfected with *TP53* siRNA or control, then exposed 6 h to *Kp*MSN and treated 48 h for Nutlin-3 (10 μM). Scale bar = 0.1 mm. **H** p53-dependent apoptosis assessed by Western blot for PARP and Caspase-3 cleavage in LS513 cells transfected with *TP53* siRNA or control and treated with 5-FU and *Kp*MSN. **I** p53-independent effect of *Kp*MSN on cell cycle distribution assessed by flow cytometry in the same conditions.

We then investigated the mechanism underlying p53 downregulation. Previously, we reported that *K. pneumoniae* LPS destabilizes p53 mRNA in stromal cells [25]. However, *TP53* mRNA level was not changed in LS513 cells exposed to *Kp*MSN (Fig. 5A), in line with the absence of NF-κB activation in these cells (Supplementary Fig. S1C). Inhibiting the p53 E3 ligase MDM2 by Nutlin-3 or the proteasome by MG132 prior to exposure to *Kp*MSN prevented p53 downregulation, demonstrating that *Kp*MSN enhances its proteasomal degradation (Fig. 5B). MDM2 dependency was confirmed using siRNA-mediated knockdown (Supplementary Fig. S7A). Immunofluorescence staining confirmed reduced p53 levels without subcellular localization changes (Fig. 5C).

Since the cytotoxicity of 5-FU, irinotecan, and oxaliplatin might be partially independent of p53 activity [35, 36], we used Nutlin-3 to specifically probe p53-dependent cell death. Pre-exposure to *Kp*MSN significantly attenuated Nutlin-3–mediated p53 stabilization and activity (Fig. 5D, E). Nutlin-3 led to robust p53 accumulation and subsequent cell death, which was fully p53-dependent as confirmed by siRNA-mediated p53 knockdown (Fig. 5E–G). In contrast, pre-exposure to *Kp*MSN significantly reduced Nutlin-3–mediated killing. Upon 5-FU treatment, *Kp*MSN impaired apoptosis in a p53-dependent manner (Fig. 5H), but *Kp*MSN-induced G1/S cell cycle arrest was independent of p53 level (Fig. 5I and Supplementary Fig. S7B), in line with p21 induction by *Kp*MSN in the absence of 5-FU (Fig. 3D and Supplementary Fig. S5).

Altogether, our data demonstrate that *Kp*MSN-induced p53 degradation protects LS513 cells from p53-dependent cell death regardless of its effect on the cell cycle.

### Macrophage-derived extracellular vesicles mediate p53 inhibition

**Fig. 6:**
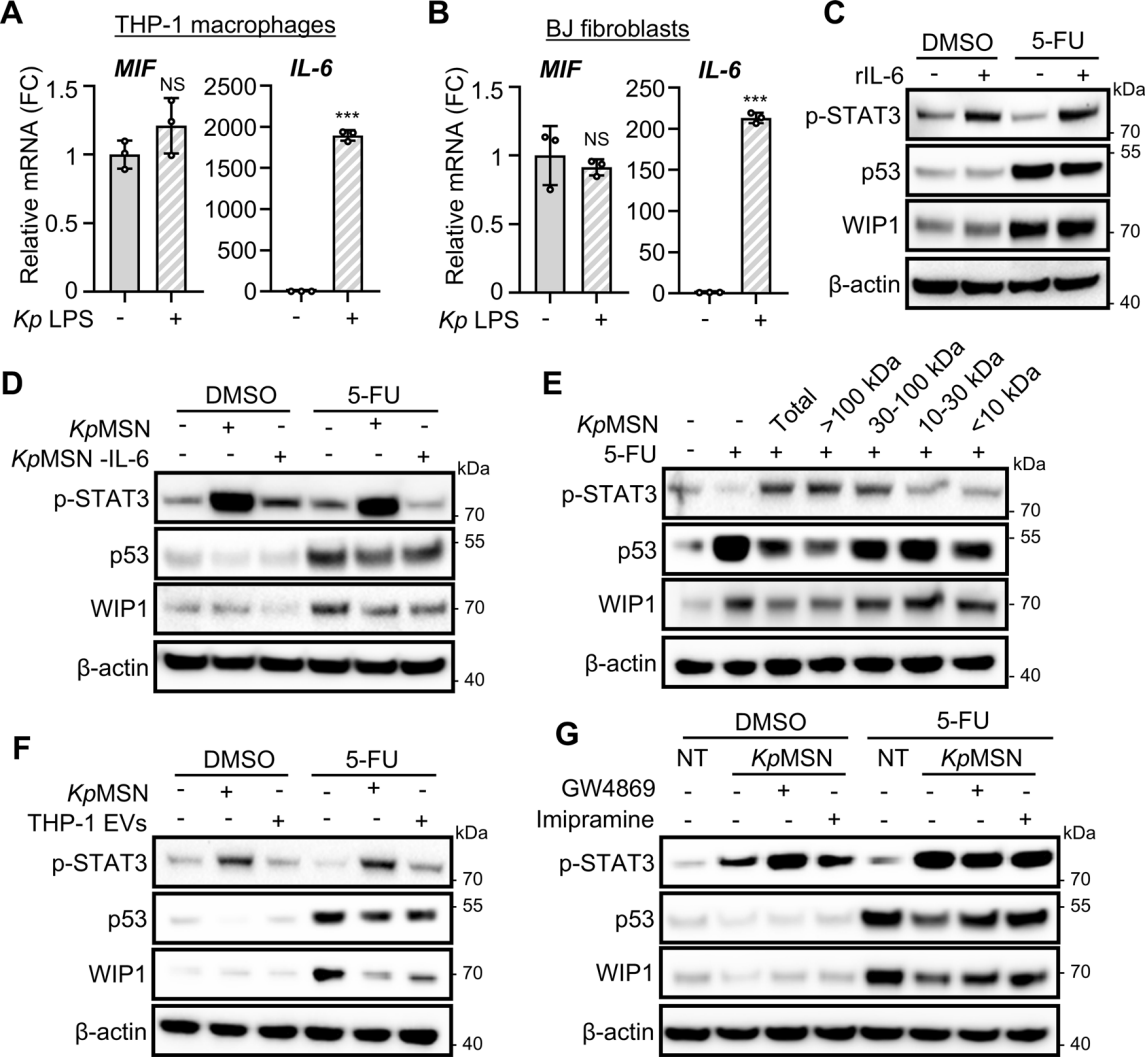
Macrophage-derived extracellular vesicles mediate p53 downregulation in colorectal cancer cells. Relative expression of *MIF* and *IL-6* in THP-1-derived macrophages (**A**) and BJ hTERT fibroblasts (**B**) following stimulation with *K. pneumoniae* LPS (*Kp* LPS), measured by RT-qPCR. **C** p53 and WIP1 levels of LS513 cells treated with recombinant IL-6 prior to 5-FU treatment, measured by Western blot. STAT3 phosphorylation is used as a positive control. **D** Similar experiment in LS513 cells exposed to *Kp*MSN depleted of IL-6 using specific antibodies (*Kp*MSN -IL-6). **E** Similar experiment in LS513 cells treated with different fractions of *Kp*MSN obtained by ultrafiltration. **F** Western blot of LS513 cells exposed to *Kp*MSN or treated with extracellular vesicles from *K. pneumoniae*-stimulated THP-1 macrophages isolated using a membrane-affinity spin column kit (THP-1 EVs). **G** Western blot of LS513 cells after exposure to *Kp*MSN from THP-1 macrophages treated with EV biogenesis inhibitors GW4869 or imipramine before LPS treatment. *p < 0.05; ***p < 0.01; NS: not significant.

p53 can be inhibited by cytokines, such as the macrophage migration inhibitory factor MIF [37] or the IL-6/STAT3 pathway [38, 39]. To assess their involvement in p53 suppression, we analyzed their expression in THP-1 macrophages and BJ hTERT fibroblasts by qPCR. While *MIF* expression remained unchanged following *K. pneumoniae* LPS stimulation, *IL-6* was strongly upregulated in both cell lines (Fig. 6A, B). However, treatment of LS513 with recombinant IL-6 could not phenocopy p53 inhibition despite activating STAT3 phosphorylation (Fig. 6C). Similarly, antibody-mediated depletion of IL-6 from *Kp*MSN did not rescue p53 inhibition or downregulation of its target WIP1 (Fig. 6D), demonstrating an IL-6/STAT3-independent mechanism.

To investigate further the mechanism of p53 downregulation, we fractionated *Kp*MSN by ultrafiltration (Fig. 6E). The p53-suppressive activity was primarily retained in the >100 kDa fraction, enriched in EVs and lipids, albeit the <10 kDa metabolite-containing fraction also had a minor effect. We then isolated EVs from *K. pneumoniae*-stimulated THP-1 macrophages using either membrane-affinity spin columns or ultracentrifugation. EV size, integrity and purity were verified by dynamic light scattering (DLS) and transmission electron microscopy (TEM) (Supplementary Fig. S8A, B). Treatment with EVs phenocopied the effect on p53, irrespective of the isolation method (Fig. 6F and Supplementary Fig. S8C). This effect was further confirmed using conditioned medium and isolated EVs from primary PBMC-derived macrophages (Supplementary Fig. S8D). Moreover, treatment of THP-1 macrophages with sphingomyelinase inhibitors (GW4869 or imipramine) to block EV biogenesis prevented the effect of the resulting *Kp*MSN on p53 in cancer cells (Fig. 6G and Supplementary Fig. S8E–H), supporting the involvement of EVs from stromal cells in p53 inhibition.

Altogether, our findings uncover a paracrine mechanism by which LPS-stimulated stromal cells attenuate p53 activity in CRC cells, thereby promoting chemoresistance in vitro. This suppression does not occur through direct cytokine signaling, but rather via EVs that drive p53 protein destabilization and functional inactivation. Notably, this mechanism selectively impairs a subset of p53 transcriptional targets correlating with treatment response, while preserving or enhancing others associated with inflammation. Our work underlines the influence of the TME in shaping p53 tumor suppressive function beyond its mutational status.

## Discussion

Our study reveals a previously unrecognized mechanism by which microbiota-driven inflammatory signaling in stromal cells suppresses p53 activity in cancer cells, contributing to chemoresistance. We demonstrate that LPS from gut-resident Enterobacteria such as *K. pneumoniae* stimulates stromal cells to secrete EVs that impair p53 protein stability and downstream signaling in CRC cells in vitro. This paracrine suppression of p53 alters the transcriptional response to genotoxic stress and compromises the cytotoxic efficacy of chemotherapeutic drugs 5-FU, irinotecan, and oxaliplatin.

Notably, inflammation does not uniformly silence p53 but affects a subset of its target genes, reshaping the transcriptional output of p53. This is in line with reports demonstrating the high context dependency and plasticity of p53 transcriptional response [40]. p53 target genes repressed by inflammation seem to better define p53 transcriptional activity in CRC cells, while others, such as p21 and TRIM22, are mostly driven by p53-independent or co-regulated pathways related to inflammation.

Albeit p53 mutations are associated with cancer progression and invasiveness, the impact of p53 functional inactivation on response to chemotherapy remains debated [17]. Most reports suggest that p53 is an important prognostic factor for response to DNA-damaging agents [41–43], but p53 mutations have also been associated with better response to treatment [44] and WT p53 with chemoresistance [45]. This paradox may come from p53 role in DNA repair, or in p53-dependent cell cycle arrest, shielding cancer cells from drug-induced apoptosis caused by replication fork collapse in S phase or by mitotic catastrophe [46, 47]. It is worth noting that missense p53 mutants often retain a partial transcriptional activity, which has been overlooked in multiple studies. In CRC, p53 mutations have no clear impact on overall survival in response to chemotherapy [33, 48]. Our data suggest that cell cycle arrest caused by bacterial-induced inflammation in the gut contributes to chemotherapy resistance via p53-independent induction of p21. However, we show that targeted activation of p53 triggers apoptosis, which is suppressed by inflammation, indicating that part of the therapeutic response is influenced by the extent of p53 activation. Overall, our results support that p53 activity in CRC, reflected by a subset of p53 targets, has a beneficial role for survival and that non-mutational inactivation of p53 via TME-derived signals might be an underappreciated contributor to poor prognosis.

The mechanistic insights presented here are primarily based on in vitro systems, allowing the deconvolution of complex variables, but which cannot fully recapitulate the cellular complexity, spatial organization, and dynamics of CRC. Analyses of patient datasets revealed correlations consistent with our findings, supporting their clinical relevance. However, future in vivo validation will be essential to define the contribution of this pathway to chemoresistance in a more physiological setting and to evaluate its potential as a therapeutic target.

While our study establishes a functional connection between inflammatory stromal cell signaling and suppression of p53 activity in CRC cells, the precise molecular mechanisms leading to p53 destabilization by MDM2 in this context remain to be fully elucidated. Interestingly, while cytokines such as IL-6 are prominently induced by LPS, they were not required for p53 suppression in our model, contrasting with previous reports [38, 39]. Our data instead support a role for EVs derived from inflamed stromal cells in mediating p53 inhibition, consistent with their recognized role in TME communication [49, 50]. Notably, EVs from tumor-associated macrophages (TAMs) have been implicated in chemoresistance in ovarian and pancreatic cancer [51, 52]. Comprehensive profiling of EV content, including proteins, lipids, and microRNAs, will be required to identify the specific EVs cargo responsible for inhibiting p53, and whether they are therapeutically targetable.

In summary, our work unveils a bacteria-instructed mechanism of chemoresistance driven by inflammation-induced suppression of p53 mediated by EVs from stromal cells. These findings highlight the importance of considering the microbial and inflammatory context of CRC when evaluating p53 function. Restoring p53 activity in the context of paracrine suppression may offer novel avenues to overcome resistance in p53 WT cancers. Therapeutic strategies targeting microbiota composition, inflammatory signaling, or EV biogenesis may thus enhance the efficacy of standard-of-care chemotherapies in CRC.

## Material and methods

### Cell culture

BJ hTERT fibroblasts were obtained from Agami’s lab [53]; THP-1 monocytic leukemia cells, LS513, SK-CO-1, RKO and SW480 colorectal cells from ATCC. Cells were cultured in DMEM (BJ hTERT, SK-CO-1, RKO) or RPMI-1640 (LS513, THP-1, SW480), supplemented with 10% FBS (Gibco), 2 mM L-glutamine, 100 U/mL penicillin, and 100 µg/mL streptomycin. THP-1 monocytes were maintained between 0.1 and 1 × 10^6^ cells/mL and were differentiated to macrophages by 20 ng/mL PMA (Sigma-Aldrich) for 24 h, then rested 24 h without PMA prior to the experiment. Mycoplasma contamination was monthly tested using the MycoAlert Detection Kit (Lonza). All experiments were performed within 10 passages from frozen stocks.

### Cell treatments

Cells were treated for 8 h (for RNA) or 16 h (for proteins) with IC50 concentration of drugs (from Sigma) 5-FU (LS513: 1 μM; SK-CO-1: 10 μM; RKO: 2 µM, SW480: 10 μM), irinotecan (5 μM) or oxaliplatin (1 μM). DMSO was used as a control. For investigating p53 proteasomal degradation, cells were pre-treated for 2 h with 10 μM MG132 or 5 μM Nutlin-3 (Sigma) prior to MSN addition. For recombinant IL-6 (#GF338, Sigma) experiments, cells were treated at 50 ng/mL for 6 h before 5-FU addition. For siRNA experiments, ON-TARGETplus siRNA SMARTpool for *TP53*, non-targeting pool (Dharmacon) or MDM2 siRNA (ThermoFischer #s8630) was transfected 24 h before treatment using Lipofectamine RNAiMAX (Invitrogen) according to the manufacturer’s instructions.

### MSN preparation

100 ng/mL of purified LPS from *K. pneumoniae* (Sigma), *E. coli* (Sigma) or *B. fragilis* was used to stimulate THP-1-derived macrophages for 8 h. For *Kp*SN treatment, a 1:50 dilution of *K. pneumoniae* culture supernatant was used. For TLR4 inhibition, THP-1 cells were pretreated with 10 μM TAK-242 (Sigma) for 1 h prior to LPS stimulation. Conditioned macrophage supernatant was filtered (0.22 µm) and added to CRC cells at 50% dilution for 6 h prior to treatment. Similarly, direct LPS pretreatment of cancer cells was performed at 100 ng/mL for 6 h prior to treatment. For IL-6 depletion, MSN was incubated with IL-6 antibody- or IgG-coated Dynabeads (Thermo Fisher) for 30 min at RT. For fractionation, filtered MSN was centrifuged through 100, 30 and 10 kDa molecular weight cutoff concentrators (Millipore). For EVs biogenesis inhibition, THP-1 cells were pretreated for 1 h with 10 µM GW4869 or 25 µM imipramine (Sigma) prior to LPS stimulation.

### EV isolation and characterization

EVs were purified either using the exoEasy Maxi Kit (Qiagen), according to the manufacturer’s instructions, or by two rounds of ultracentrifugation at 100,000 × *g* for 2 h at 4 °C, separated by a washing step of the pellet with PBS. EV protein concentrations were quantified by Qubit assay (Thermo Fisher) and stored at –80 °C until further use. For treatment, EVs were used at a concentration of approximately 100 μg of protein for 6 h prior to treatment.

EV size distribution was evaluated by DLS on a Panalytical Zetasizer Ultra instrument (Malvern) and analyzed using Zetasizer ZS Xplorer (Malvern). To assess morphology and purity, EVs were adsorbed onto carbon-coated copper grids, negatively stained with uranyl acetate and visualized by TEM on a Hitachi HT7800 equipped with a 20 MPx Xarosa camera. For lipid quantification, EVs were incubated with FM4-64 (2.25 μg/mL) at 37 °C for 10 min. Samples were excited at 485 nm, and fluorescence was measured at 670 nm on a Tecan Infinite MPlex.

### Cell viability assays

10,000 cells/well were seeded in 96-well plates two days prior to treatment, pre-incubated with MSN for 6 h, then treated as indicated for 40 h. Resazurin (0.15 mg/mL; Thermo Fisher) was then added for 4 h, and fluorescence was measured at 560 nm/590 nm (excitation/emission) using an Infinite® M Plex plate reader (Tecan).

### Immunoblotting and qPCR

Western blots and qPCR were performed as previously described [54]. More details are provided in Supplementary methods. All uncropped Western blots are available in Supplementary material.

### LC-MS/MS

Following treatment, cells were washed twice with PBS and lysed in 8 M urea, 1% SDS, 50 mM Tris at pH 8.5 with protease inhibitors (Thermo Fisher). Sample preparation and processing were performed according to our previous studies [55, 56]. More details are provided in Supplementary methods. Gene Ontology (GO: biological process) and pathway enrichment analysis were performed using g: Profiler [57] and visualized using Cytoscape (v3.6.1) [58] as described by Reimand et al. [59]. The cut-off for differentially abundant proteins was *p*-value < 0.05 and |log₂FC | > 0.5. GSEA was performed using GSEA software (https://www.gsea-msigdb.org/gsea/index.jsp) [60]. The complete lists of differentially abundant proteins and enriched GO terms upon *Kp*MSN treatment are provided in Supplementary Table S1.

### Cell cycle analysis

Cells were detached using TrypLE™ Express Enzyme 1X (Gibco), resuspended in PBS 2 mM EDTA, incubated 3 min on ice, washed twice, and fixed in 70% ethanol for 2 h at –20 °C. Cells were then treated with RNAse A (100 μg/mL, 20 min, 37 °C), and stained with propidium iodide (5 μg/mL, 10 min). Flow cytometry was performed using a LSR Fortessa Flow Cytometer (BD Biosciences) and analyzed with FlowJo 10 (BD Biosciences).

### TCGA patient data analysis

Clinical data, normalized RNAseq expression data and *TP53* mutations from colorectal adenocarcinoma (COAD) patients were extracted from The Cancer Genome Atlas (TCGA; https://www.cancer.gov/tcga) using cBioPortal (http://cbioportal.org) [61, 62]. Pearson-based unsupervised hierarchical clustering of differentially (*p* < 0.05) abundant p53 targets from the Fisher gene set [29] in our proteomics data was performed using R. The gene list for each cluster is available in Supplementary Table S2. Signature expression scores were calculated by ssGSEA [60, 63] using the Python package gseapy. Inflammation and TIL scores were calculated by ssGSEA using the gene set Hallmark_Inflammatory_Response (M5932) from MSigDB and genes defined by Truntzer et al. [32], respectively. High and low inflammation groups were defined by the 10% tumor with the highest and lowest inflammation scores. For survival analysis, only patients receiving 5-FU, oxaliplatin or irinotecan (including in combination) were analyzed. Due to the low number of patients in the studied group (*n* = 49), tumors were divided into two groups for considering high and low Cluster 1 and 2 signatures.

### Statistical analysis

Correlation analyses were performed using the Pearson correlation coefficient. For survival analysis, significance was calculated using logrank Mantel–Cox test. Otherwise, statistical significance was calculated using a two-tailed Student *t* test from at least three independent experiments. *p < 0.05; ***p < 0.01.

## Supplementary information

Supplementary Figures

Supplementary Table S1

Supplementary Table S2

Supplementary Methods

Uncropped Western blots

## Data Availability

The MS proteomics data have been deposited to the ProteomeXchange Consortium via the PRIDE partner repository (http://www.ebi.ac.uk/pride) [64] with the dataset identifier PXD064521.
