# Supplementary material for: Klebsiella pneumoniae LPS drives stromal-mediated repression of p53 and colorectal cancer chemoresistance
Source: Cell Death Dis. 2026 Apr 20;17(1):395. doi: 10.1038/s41419-026-08756-4 (PMC13092637; doi:10.1038/s41419-026-08756-4)
Supplement: Supplementary file 1 — Supplementary Figures [file 41419_2026_8756_MOESM1_ESM.pdf]

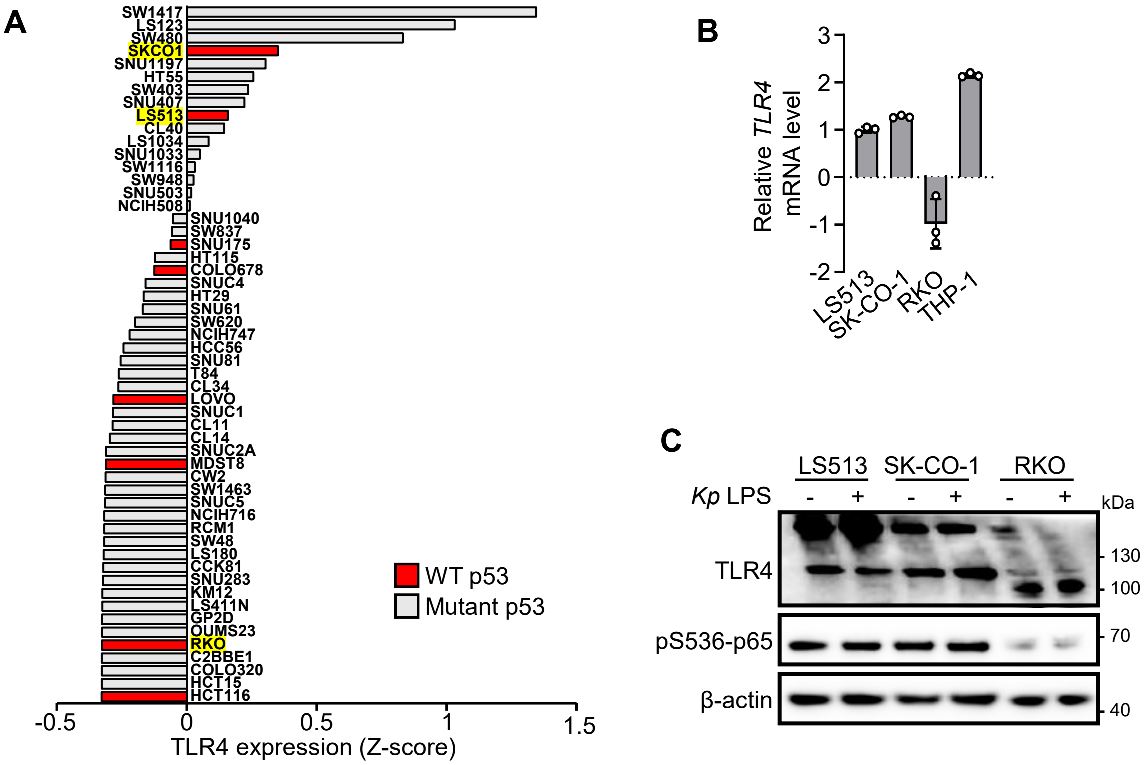

Supplementary Figure S1. (A) RNAseq data showing relative *TLR4* expression level of 54 CRC cell lines from the Broad Institute Cancer Cell Line Encyclopedia[26]. CRC cells used in this study are highlighted. **(B)** Basal TLR4 expression level measured by qPCR in three different cancer cell lines compared to THP-1-derived macrophages. **(C)** Western blot showing TLR4 protein level and phosphorylation of NF-κB subunit p65 upon exposure to *Kp* LPS.

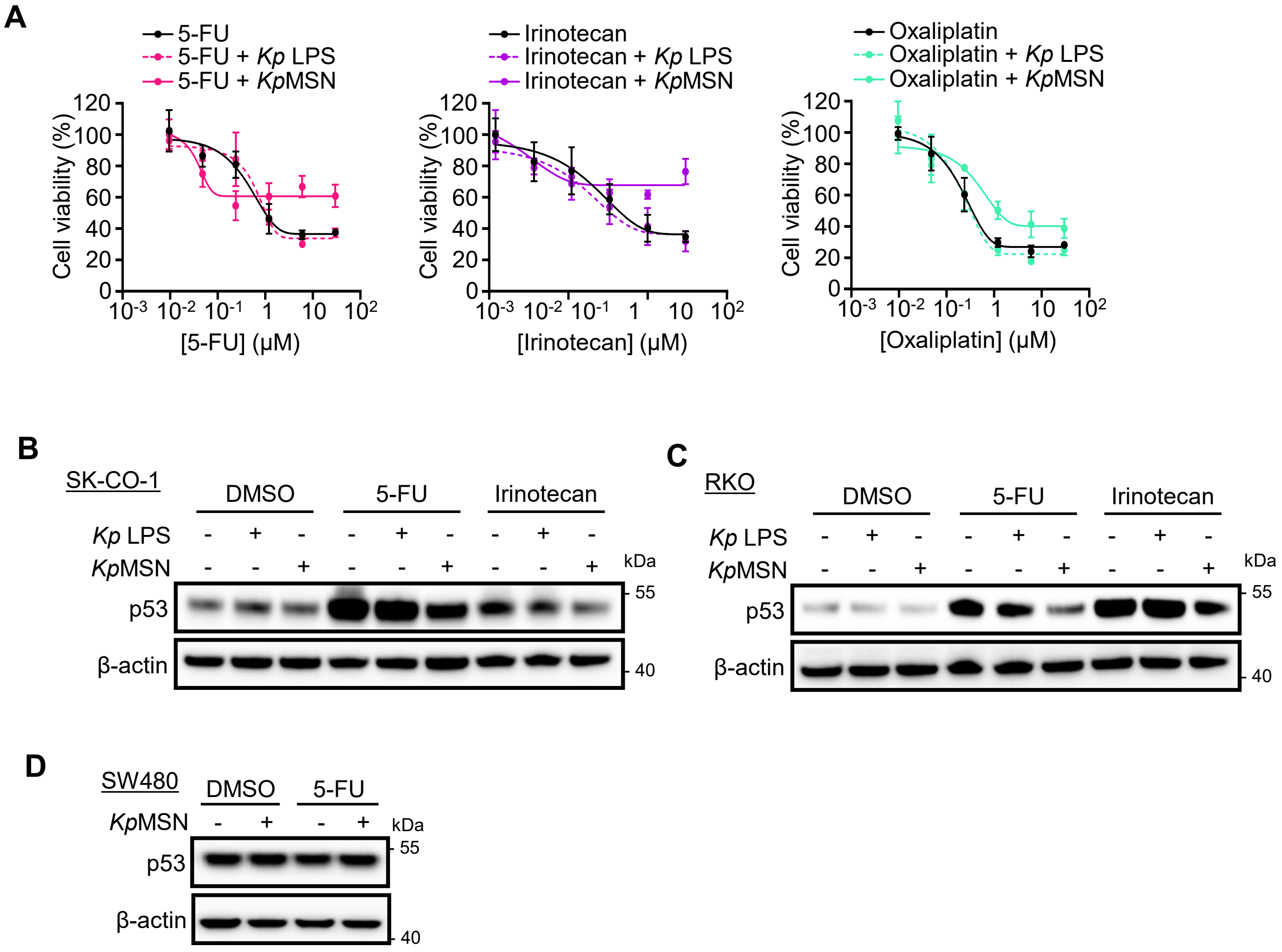

Supplementary Figure S2. (A) Cell viability of LS513 cells treated with 5-FU, irinotecan or oxaliplatin alone or after exposure to *Kp*MSN or *Kp* LPS, measured by resazurin assay. **(B-C)** Western blot analysis of p53 protein level in WT p53 colorectal cancer cell lines SK-CO-1 (C) and RKO (C), treated with 5-FU or irinotecan after exposure to *Kp* LPS or *Kp*MSN. **(D)** Western blot analysis of p53 protein level in mutant p53 colorectal cancer cell line SW480 treated with 5-FU after exposure to *Kp*MSN.

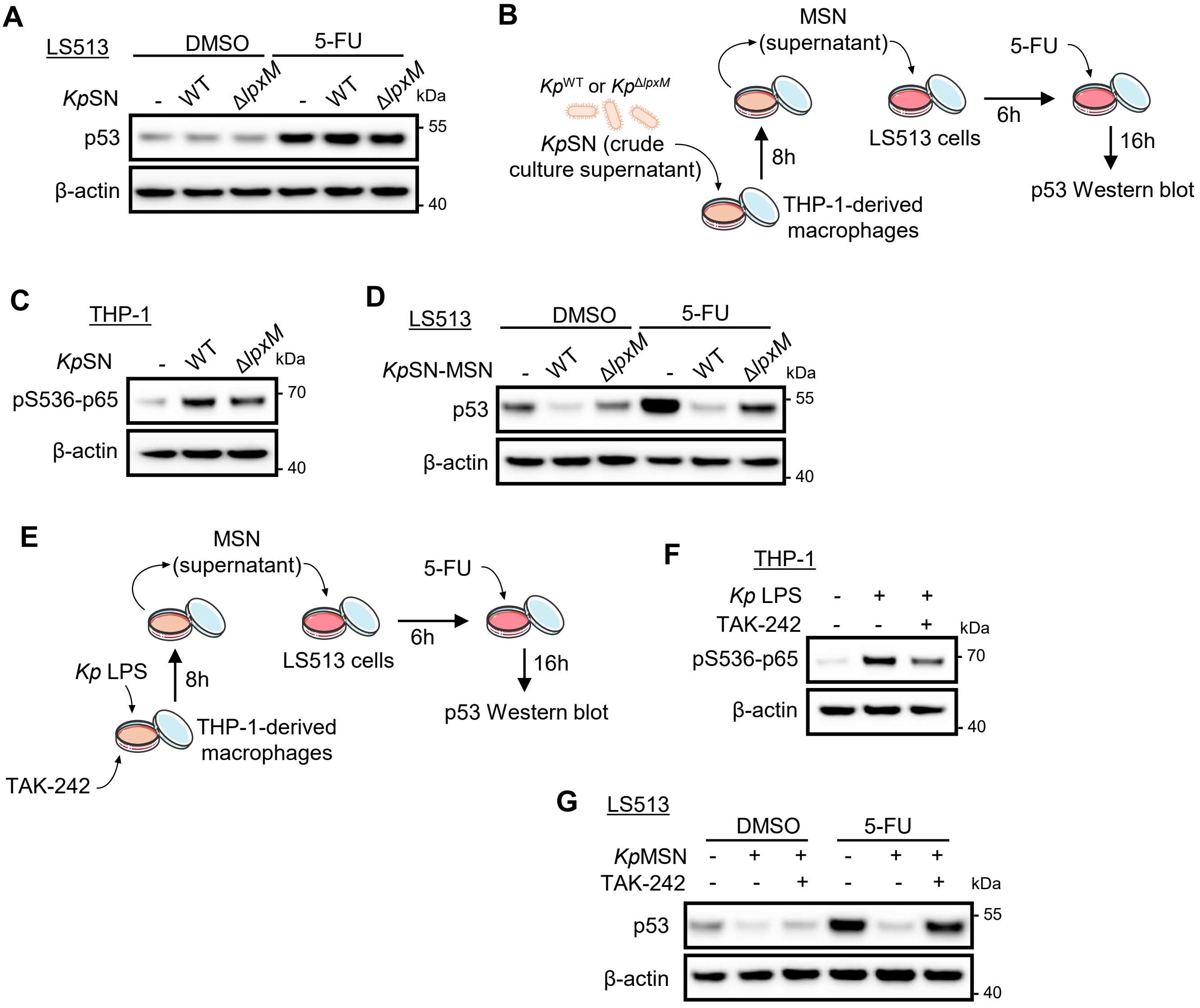

Supplementary Figure S3. (A) Western blot analysis of p53 protein level in LS513 cells pre-exposed to crude supernatant from *K. pneumoniae* culture (*Kp*SN) derived from either wild-type (WT) bacteria or LPS biosynthesis-deficient mutant (Δ*lpxM*), followed by 5-FU treatment. **(B)** Experimental workflow used to generate conditioned medium from *Kp*SN-stimulated THP1 cells (*Kp*SN-MSN) and subsequent LS513 cells treatment. **(C)** NF-κB activation in THP-1 cells following *Kp*SN stimulation, assessed by Western blot analysis of phosphorylated p65. **(D)** Western blot analysis of p53 protein level in LS513 cells exposed to *Kp*SN-MSN generated from WT or Δ*lpxM K. pneumoniae*. **(E)** Experimental workflow used to generate *Kp*MSN upon pharmacological inhibition of TLR4 in THP-1 cells prior to treatment of LS513 cells. **(F)** Validation of TLR4 inhibition in LPS-stimulated THP-1 cells, assessed by Western blot analysis of p65 phosphorylation following treatment with the TLR4 inhibitor TAK-242. **(G)** Western blot analysis of p53 protein level in LS513 cells exposed to *Kp*MSN from TAK-242 treated THP-1 cells.

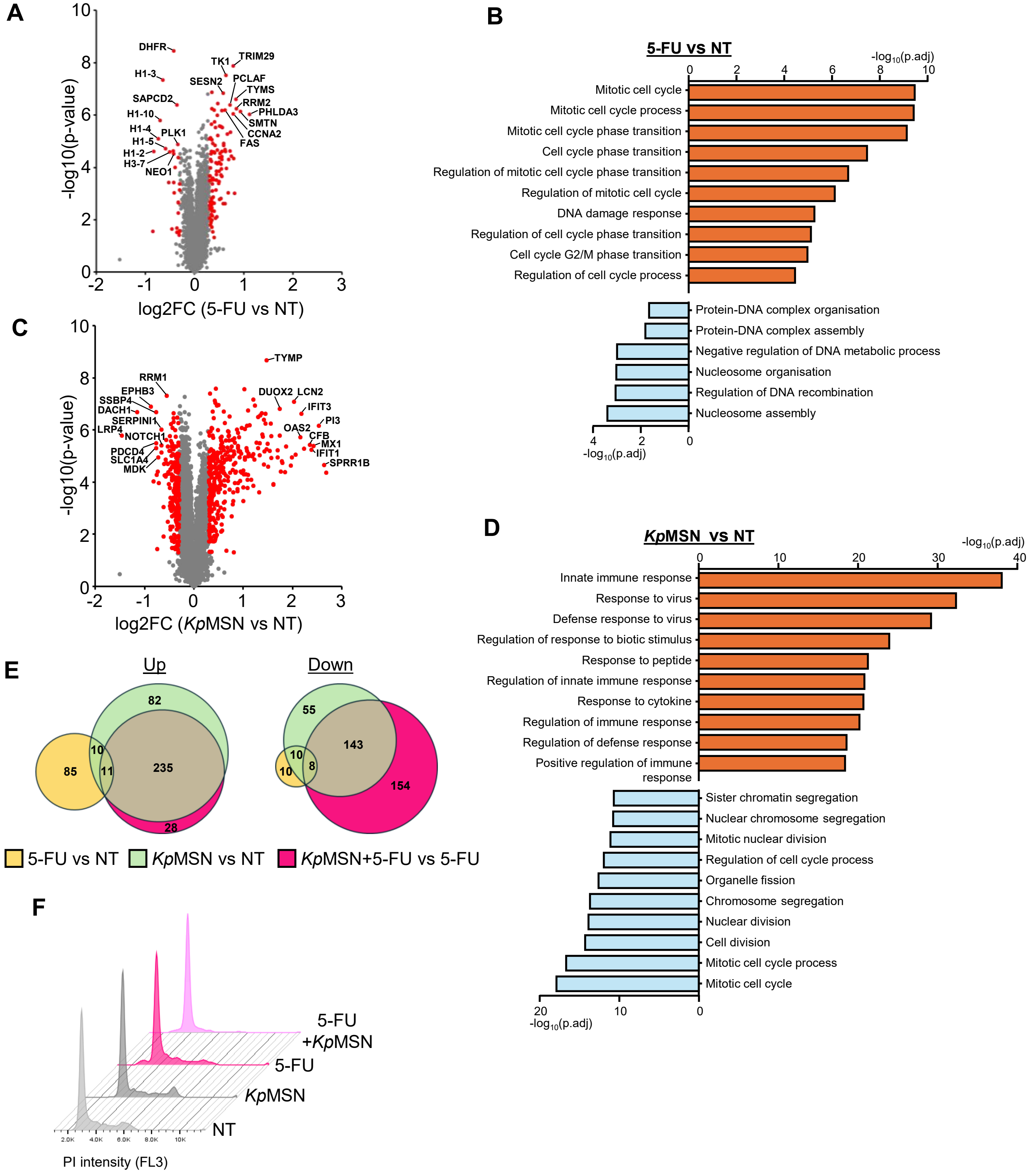

Supplementary Figure S4. LC-MS/MS performed on LS513 cells treated with vehicle (NT), 5-FU and *Kp*MSN. (**A**) Volcano plot comparing 5-FU versus NT. Top differentially abundant proteins are indicated. (**B**) Top up- and downregulated pathways in Gene Ontology (GO) analysis of differentially abundant proteins upon treatment with 5-FU. (**C-D**) Similar analysis comparing *Kp*MSN versus NT. (**E**) Venn diagrams showing up- and downregulated protein overlap between the different conditions. (**F**) Cell cycle profile of LS513 cells upon exposure to *Kp*MSN, treatment with 5-FU or combination, obtained by flow cytometry after propidium iodide (PI) staining.

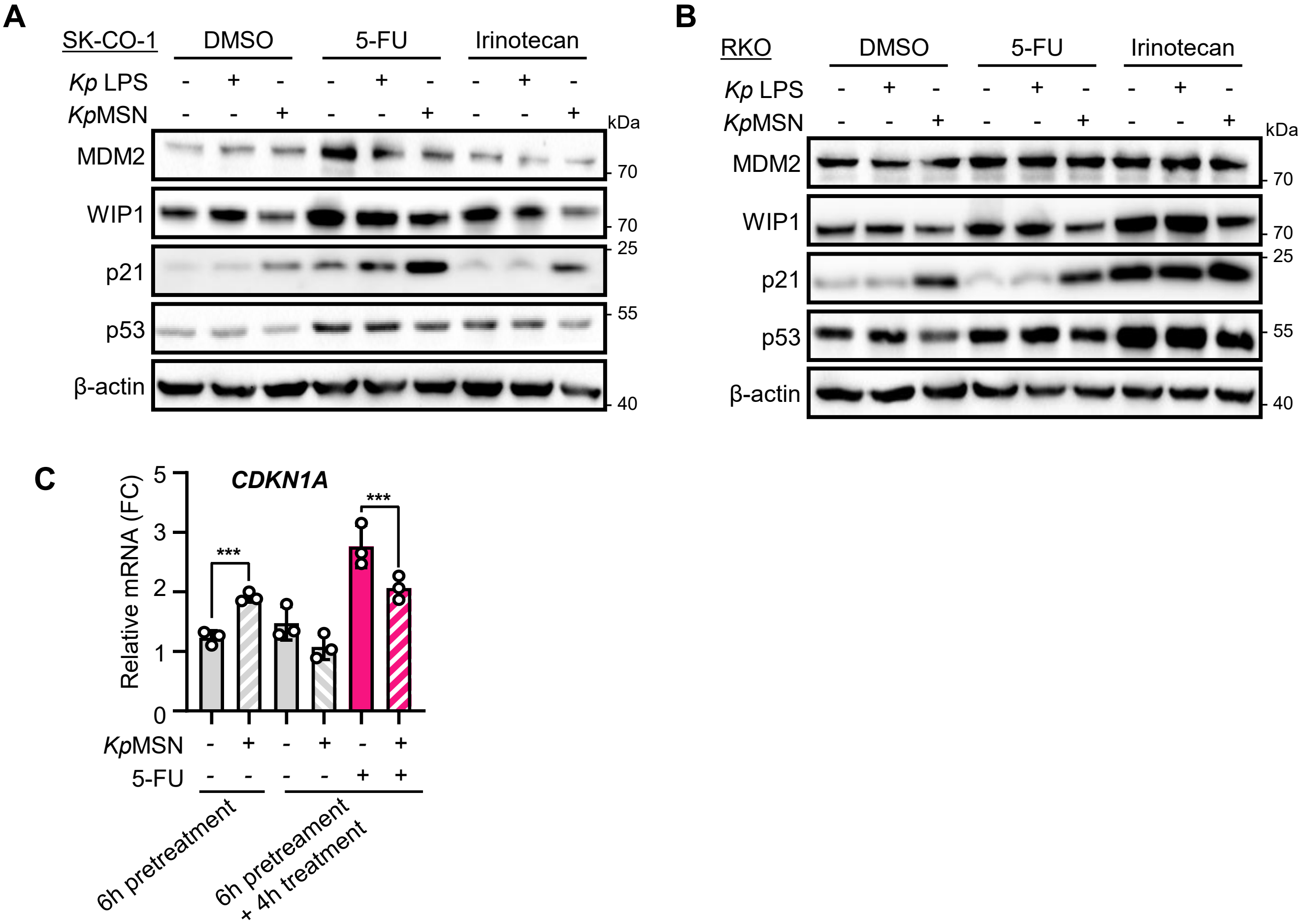

Supplementary Figure S5. (A-B) Level of direct p53 targets MDM2, WIP1 and p21 assessed by Western blot in SK-CO-1 (A) and RKO (B) colorectal cancer cells treated with 5-FU or irinotecan after exposure to *Kp* LPS or *Kp*MSN. **(C)** qPCR for *CDKN1A* expression upon pretreatment by *Kp*MSN at early time point (6h) or after 4h of subsequent treatment with 5-FU. *** $p<0.01$.

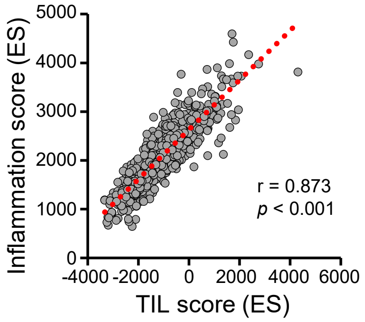

Supplementary Figure S6. Correlation of inflammation scores with tumor-infiltrating lymphocyte (TIL) scores in the TCGA PanCancer Atlas Colorectal Adenocarcinoma cohort.

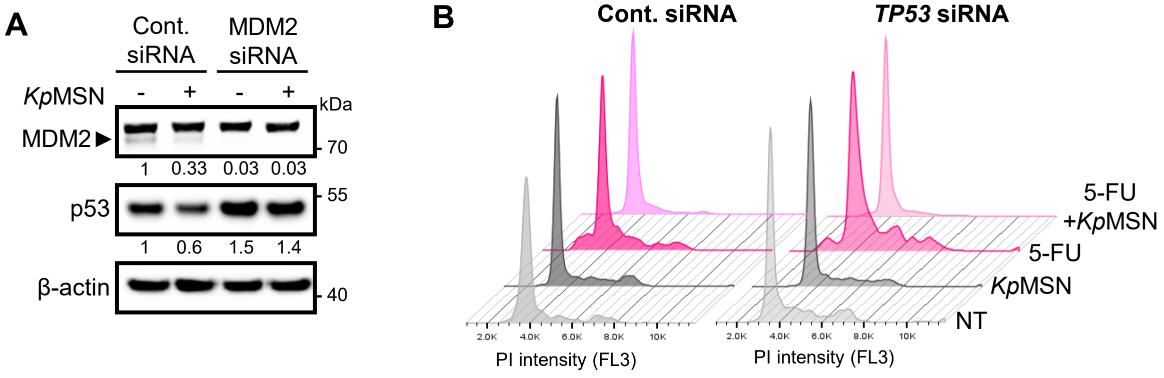

Supplementary Figure S7. (A) MDM2 and p53 protein level measured by Western blot in LS513 cells upon MDM2 silencing. The densitometric quantification of the bands normalized to β-actin is indicated under each blot. **(B)** Cell cycle profiles of LS513 cells transfected with *TP53* siRNA or control (Cont. siRNA), upon exposure to *Kp*MSN, treatment with 5-FU or combination, obtained by flow cytometry after propidium iodide (PI) staining.

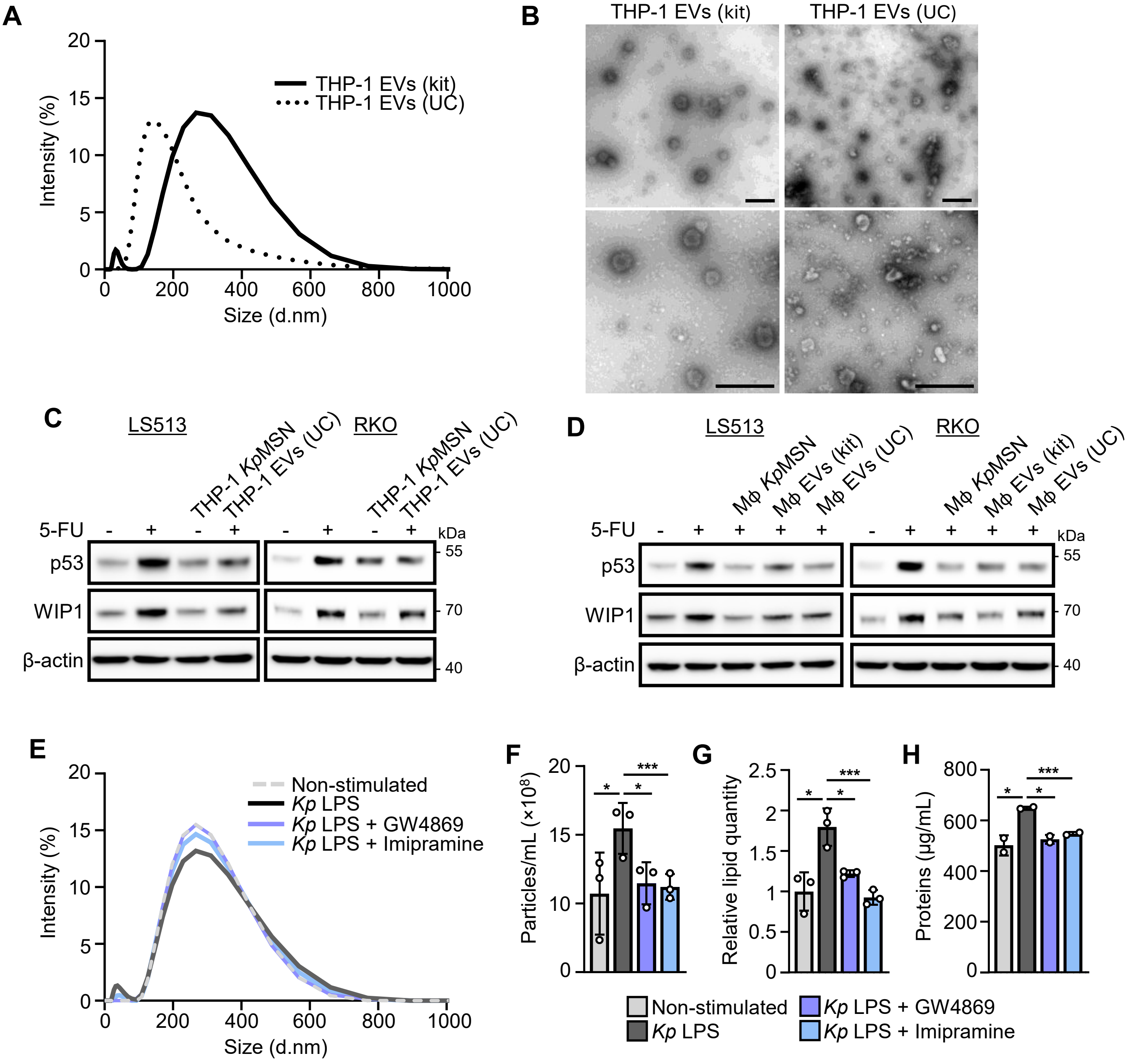

Supplementary Figure S8. (A) Size distribution of extracellular vesicles (EVs) isolated either using a membrane-affinity spin column–based kit or by ultracentrifugation (UC), analyzed by dynamic light scattering (DLS) **(B)** Morphology, integrity, and purity of EVs isolated by kit or UC, assessed by transmission electron microscopy. Scale bar = 500 nm. **(C)** Western blot analysis of LS513 and RKO cells exposed to *Kp*MSN or treated with UC-isolated EVs from *Kp* LPS-stimulated THP-1 macrophages. **(D)** Western blot analysis of LS513 and RKO cells exposed to *Kp*MSN or purified EVs, both generated by *Kp* LPS-stimulated PBMC-derived primary macrophages (Mϕ). **(E-F)** Size distribution and particle concentration of EVs produced by *Kp* LPS-stimulated THP-1 macrophages upon treatment with GW4869 or imipramine. **(G-H)** Quantification of total lipid (G) and protein (H) content in EVs isolated from THP-1 macrophages upon treatment with GW4869 or imipramine. * $p<0.05$; *** $p<0.01$.

Gene	Forward primer sequence	Reverse primer sequence
ACTB	AGAGCCTCGCCTTTGCCGAT	TTGCACATGCCGGAGCCGTT
CDKN1A	CTGTCACTGTCTTGTACCC	AGTGGTAGAAATCTGTCATGC
COQ8A	GGCTACGAGGTCAAGGTGAG	CCCCCTGTGCTTTCATCCAT
GAPDH	AGCCAAATTCGTTGTCATAC	GGTCTCCTCTGACTTCAACA
IL6	CACACAGACAGCCACTCACC	TTTTCTGCCAGTGCCTCTTT
MDM2	GGCAGGGGAGAGTGATACAG	GAAGCCAATTCTCACGAAGG
MIF	CTGCACAGCATCGGCAAGAT	AGTTGATGTAGACCCTGTCCG
PPM1D	TTGTCAGAGCTGTGGAGGTG	CGATTCACCCCAGACTTGTT
RPL13A	CCTGGAGGAGAAGAGGAAAGAGA	TTGAGGACCTCTGTGTATTTGTCAA
SESN1	ATTCGGCTGTGGAATCAGTC	TCCACACTGTGATTGCCATT
TLR4	ATGCCCAGAGAGGACGAGTA	CATGCACCAAAAGCTTAGCA
TP53	TTTGGGTCTTTGAACCCTTG	CCACAACAAAACACCAGTGC
TRIM22	AGGTTGAGGGGATCGTCAGT	TGGCTTCTTCAATGTCCAGC
ZMAT3	GAATGAGCAATGTGGTCGAG	GGAACTGAAGGAGGCATCAC

Supplementary Table S3. qPCR primers.
