# Supplementary material for: Klebsiella pneumoniae LPS drives stromal-mediated repression of p53 and colorectal cancer chemoresistance
Source: Cell Death Dis. 2026 Apr 20;17(1):395. doi: 10.1038/s41419-026-08756-4 (PMC13092637; doi:10.1038/s41419-026-08756-4)
Supplement: Supplementary file 4 — Supplementary Methods [file 41419_2026_8756_MOESM4_ESM.pdf]

Supplementary Methods

Bacteroides fragilis LPS isolation

B. fragilis was cultured anaerobically in brain heart infusion (BHI) broth at 37°C until late log phase. Bacterial cells were harvested by centrifugation at 5,000 × g for 15 min at 4°C, washed twice with PBS, and resuspended in endotoxin-free water. LPS was extracted by CD BioGlyco using hot phenol-water method. Briefly, bacterial pellets were mixed with an equal volume of preheated 90% phenol (pH 6.6) and incubated at 68°C for 15 min with vigorous stirring. The mixture was cooled on ice and centrifuged at 10,000 × g for 30 min at 4°C. The aqueous phase was collected, and the phenol phase was re-extracted with water. Crude LPS preparations were treated with 10 µg/mL DNase I (Merck), 10 µg/mL RNase A (Merck), and 100 µg/mL proteinase K (Sigma) for 2 h at 37°C, followed by phenol/chloroform/isoamyl alcohol extraction (25:24:1) and dialysis. The purified LPS was lyophilized and stored at -80°C.

Klebsiella pneumoniae supernatant (KpSN) preparation

WT and Δ*lpxM* mutant of *K. pneumoniae* (previously described[25]) were cultured in DMEM at 37°C, 200 rpm until an optical density (OD_{600}) of 0.8. Bacterial cultures were centrifuged at 4000 rpm for 10 min. Supernatants were filtered on 0.22 µm filter (Filtropur S, Sarstedt) and freezed at -20°C until further use.

PBMC-derived macrophages preparation

For experiments on PBMC-derived macrophages, monocytes were isolated from anonymous buffy coats of healthy blood donors (Karolinska University Hospital) using Ficoll gradient and centrifugation. Briefly, blood was diluted in PBS and layered on to Ficoll-Paque (GE Healthcare) and centrifuged at 1,200 rpm for 20 min. The interface layer containing the monocytes was collected and monocytes were further washed twice in PBS. Monocytes were then incubated 2 h for adhesion in RPMI 1640 and unattached cells were washed with PBS. For differentiation into macrophages, monocytes were cultured in RPMI 1640, 2mM I-glutamine, 10% FBS, streptomycin/penicillin (Sigma-Aldrich) supplemented with increasing concentration of Granulocyte-Macrophage Colony-Stimulating (Sigma; up to 400 ng/ml) for 7 days.

Western blots

Total cell lysates were extracted using ice cold RIPA buffer (Thermo Fisher) supplemented with cOmplete protease and PhosSTOP phosphatase inhibitors (Roche). Samples were denatured in Laemmli buffer at 95°C for 5 min and separated by SDS-PAGE. For detection of apoptotic markers, both adherent and floating (dead) cells were collected and lysed together. Western blot signals were detected by chemiluminescence using a ChemiDoc Imaging System (Bio-Rad).The following antibodies were used for immunoblotting: p21

(#610233; BD Transduction); p53 (sc-126; Santa Cruz); phospho-STAT3 (#9145; Cell Signaling); phospho-NF-κB p65 (Ser536) (#3031; Cell Signaling); TLR4 (sc-293072, Santa Cruz); WIP1 (sc-376257; Santa Cruz); MDM2 (OP115; Merck); cleaved PARP1(#5625; Cell Signaling); cleaved Caspase-3 (#9661; Cell Signaling); cyclin B1(sc-245; Santa Cruz); cyclin E1 (#4129; Cell Signaling); cyclin D1 (#2922; Cell Signaling); β-actin (#MAB-1501; Millipore).

Fluorescence microscopy

Immunofluorescence was performed using anti-p53 (sc-126; Santa Cruz) and Alexa Fluor 488–conjugated secondary antibody (Thermo Fisher). Images were acquired with a Zeiss LSM800 Airyscan confocal microscope.

Quantitative real-time PCR

RNA was extracted using Aurum total RNA kit (Bio-Rad), and cDNA was synthesized using iScript cDNA synthesis kits (Bio-Rad). qPCR was performed with SsoAdvanced Universal SYBRGreen SuperMix (Bio-Rad) on a CFX384 Real-Time System (Bio-Rad). qPCR primers are detailed in Supplementary Table S3. *RPL13A*, *ACTB* and *GAPDH* were used as reference genes. Data were processed using Bio-Rad CFX 3.1 using the ΔΔCT method. Error bars represent standard deviation from mean of at least three independent experiments.

LC-MS/MS sample preparation and analysis

Lysates were sonicated 1 min on ice using Branson probe sonicator and 3 s on/off pulses with a 30% amplitude. Protein concentration was measured using a BCA Protein Assay Kit (Thermo Fisher). An aliquot of 25 µg each sample was reduced with 10 mM dithiothreitol (DTT) for 1 h at room temperature, alkylated with 50 mM iodoacetamide (IAA) in the dark at room temperature and subjected to methanol chloroform precipitation. Samples were resuspended in 8M urea (pH 8.0) in 20 mM EPPS (3-[4-(2-Hydroxyethyl)piperazin-1-yl]propane-1-sulfonic acid) buffer, then diluted to 1.6M urea and digested with trypsin (1:60 trypsin:protein). Each digest was labeled with a TMTpro 16-plex reagent (Thermo Fischer). Samples were pooled and cleaned by Sep-Pack C18 columns (Waters). The pooled sample was fractionated off-line by capillary reversed-phase chromatography at pH 10 into 24 fractions and dried. LC-MS/MS analyses were performed on a Q Exactive HF hybrid quadrupole-Orbitrap mass spectrometer (Thermo Fisher). The instrument was equipped with an EASY ElectroSpray source and connected online to an Ultimate 3000 nanoflow UPLC system. Peptide separation was performed on an EASY-Spray C18 reversed-phase nano-LC column (Acclaim PepMap RSLC; length 50 cm; inner diameter 2 µm; particle size 2 µm; pore size 100 Å; Thermo Scientific) at 55 °C and a flow rate of 300 nL/min. Peptides were separated using

a binary solvent system consisting of 0.1% (v/v) formic acid (FA), 2% (v/v) acetonitrile (ACN) as solvent A and 98% ACN (v/v), 0.1% (v/v) FA as solvent B. They were eluted with a gradient of 3–26% B in 97 min, and 26–95% B in 9 min. Subsequently, the analytical column was washed with 95% B for 5 min before re-equilibration with 3% B. The mass spectrometer was operated in a data-dependent acquisition mode. A survey mass spectrum (from m/z 375 to 1700) was acquired in the Orbitrap analyzer at a nominal resolution of 120,000. The automatic gain control (AGC) target was set 3x106, with the maximum injection time of 100 ms. The 18 most abundant ions in charge states 2+ to 7+ were isolated with 1.4 Th, fragmented using HCD MS/MS with 34% normalized collision energy, and detected in the Orbitrap analyzer at a nominal mass resolution of 60,000. The AGC target for MS/MS was set 5x105 with a maximum injection time of 54 ms, whereas dynamic exclusion was set to 30 s. Proteome Discoverer 3.0 software (Thermo Scientific) with MS Amanda 2.0 search engine was utilized for the database search and quantification against the SwissProt *Homo sapiens* protein database (version 2025_02_05; 20,340 entries). Cysteine carbamidomethylation was set as fixed modification, along with TMTpro modifications (+304.207 Da), methionine oxidation, as variable modifications. Enzyme specificity was defined as trypsin with a maximum of two missed cleavages. A 1% false discovery rate was employed as filter at both the protein and peptide levels using Percolator. Contaminants were removed, and proteins with missing values were eliminated. The quantified abundance of each protein in each sample (labeled with a different TMTpro reagent) was normalized to the total intensity of all proteins in that sample. For each protein, the average normalized protein abundance in the treated replicates was divided by the average normalized abundance of that protein in the vehicle-treated replicates. The average ratio across replicates of each treatment compared to control was calculated, and the Log2 values of these ratios were determined. p-values were calculated using two-tailed Student's t-test.
