# Supplementary material for: Klebsiella pneumoniae LPS drives stromal-mediated repression of p53 and colorectal cancer chemoresistance
Source: Cell Death Dis. 2026 Apr 20;17(1):395. doi: 10.1038/s41419-026-08756-4 (PMC13092637; doi:10.1038/s41419-026-08756-4)

Fig.1B

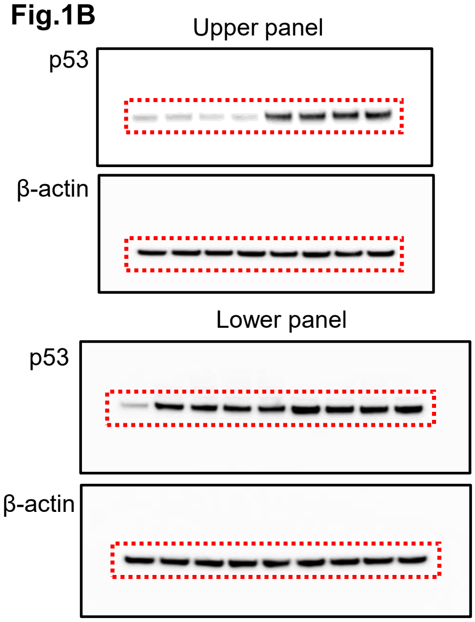

Fig.1D

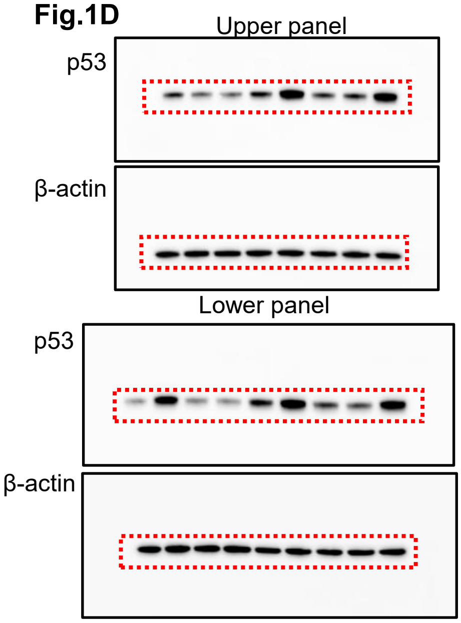

Fig.1F

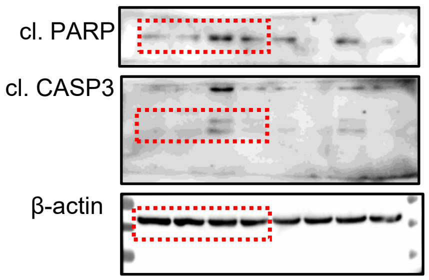

Fig.1E

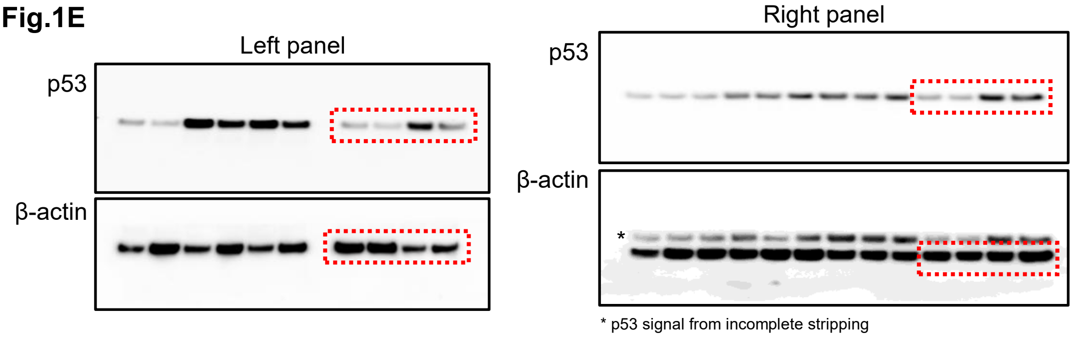

Fig.2G

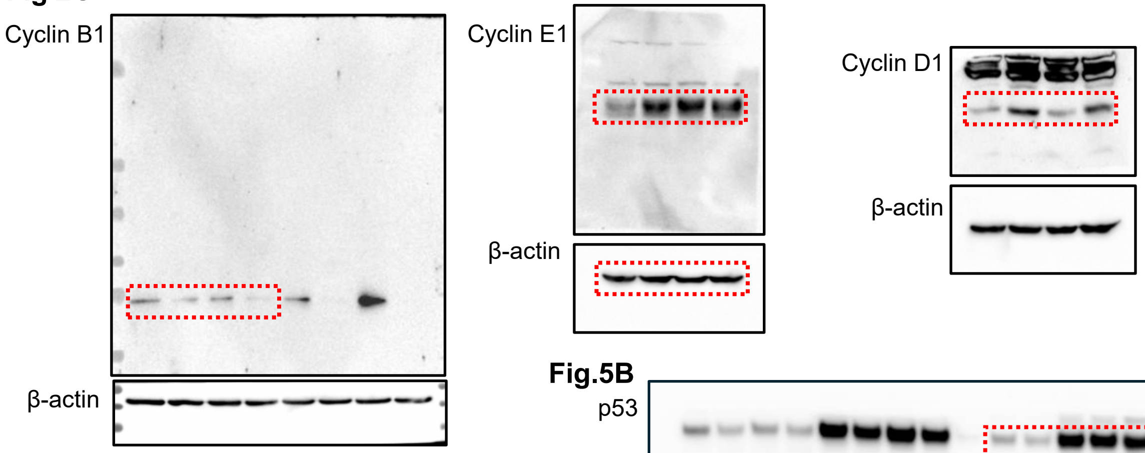

Fig.3D

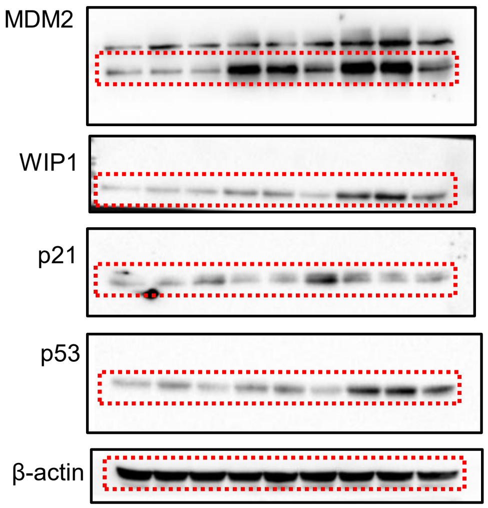

Fig.5B

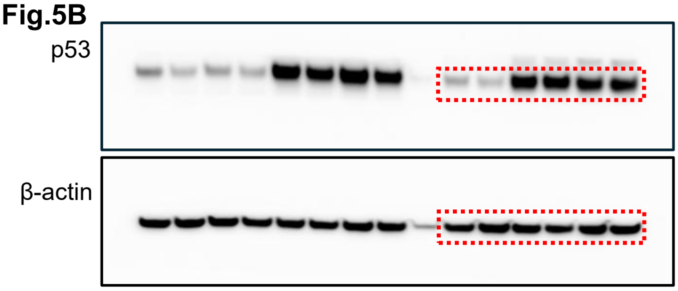

Fig.5D

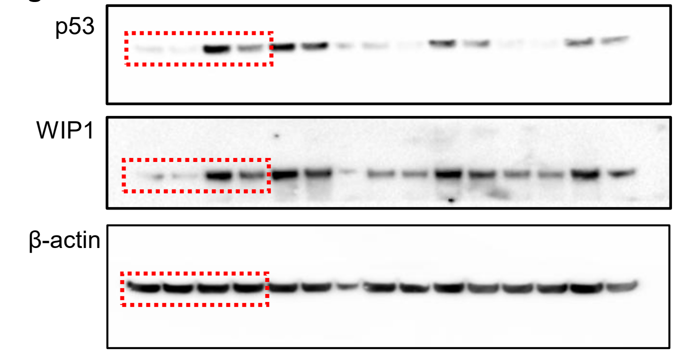

Fig.5E

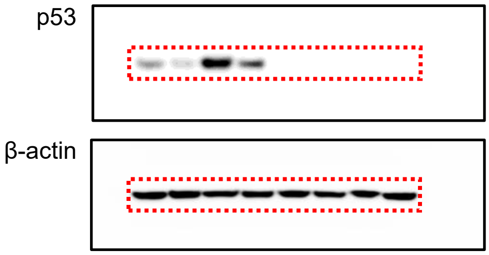

Fig.5H

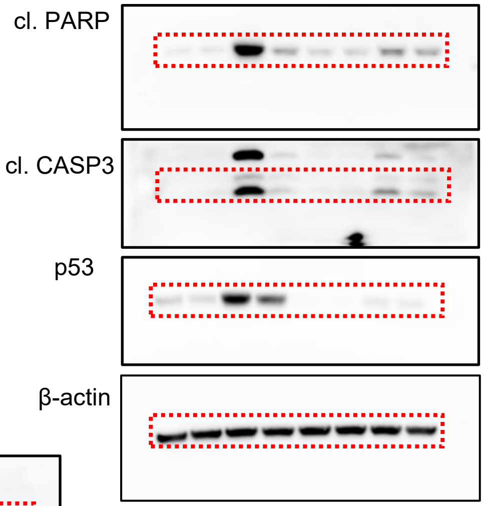

Fig.6C

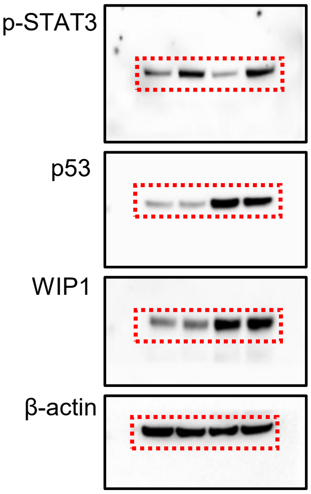

Fig.6D

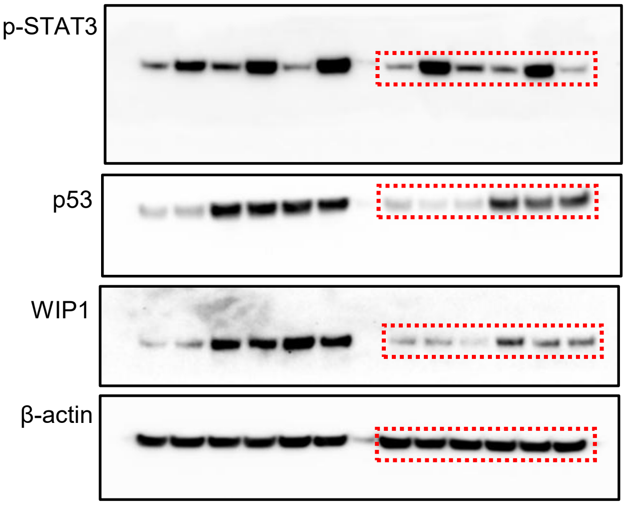

Fig.6E

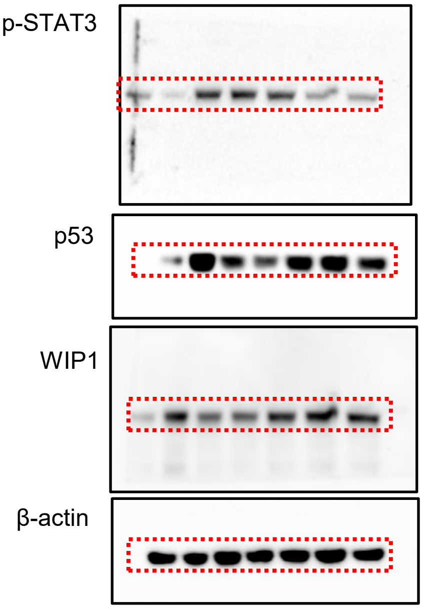

Fig.6F

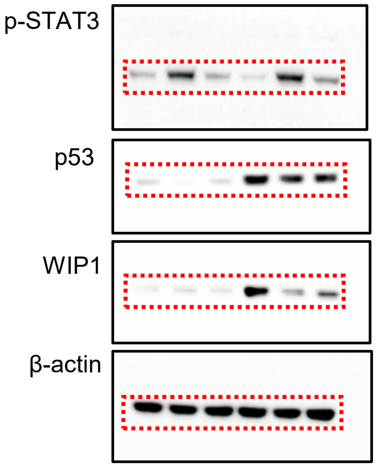

Fig.6G

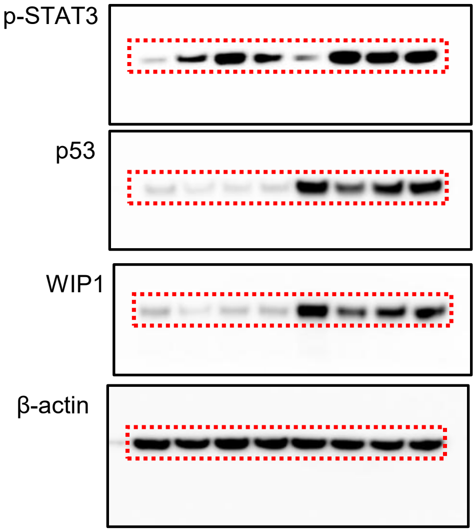

Sup. Fig.S1B

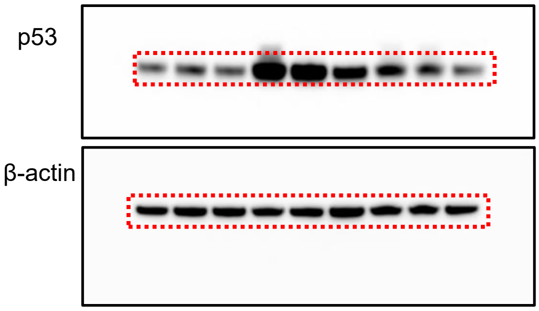

Sup. Fig.S1C

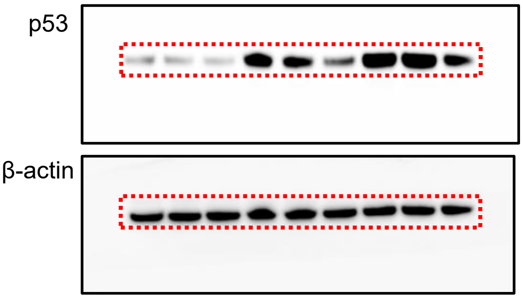

Fig.6C

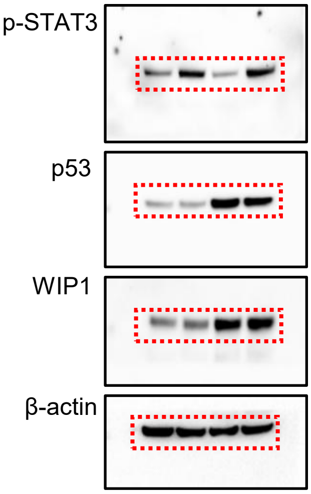

Fig.6D

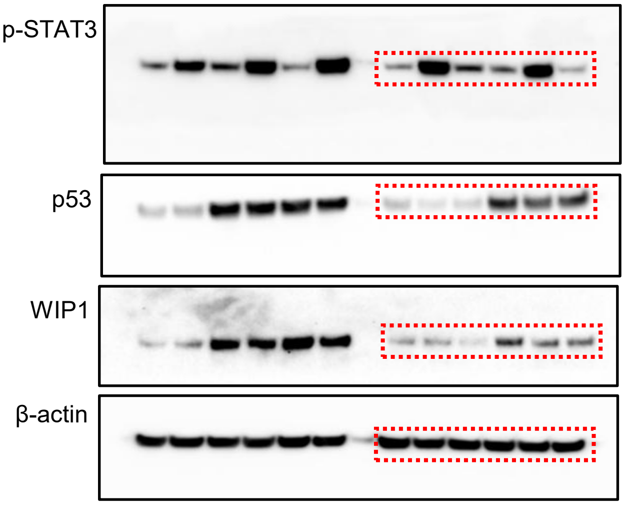

Fig.6E

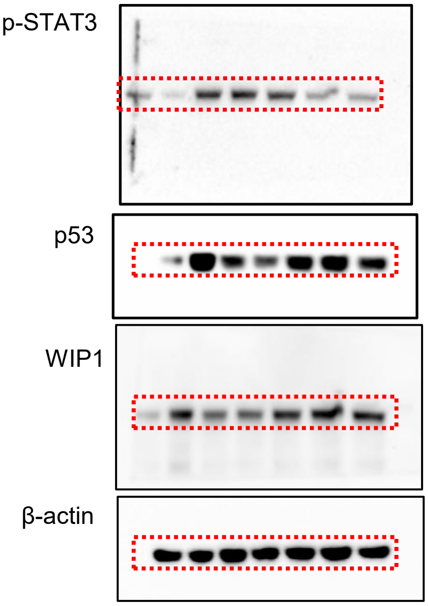

Fig.6F

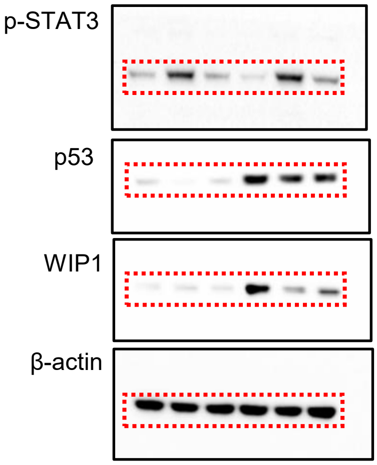

Fig.6G

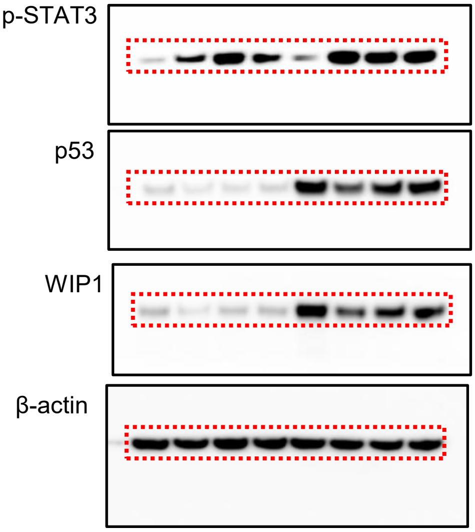

Sup. Fig.S1C

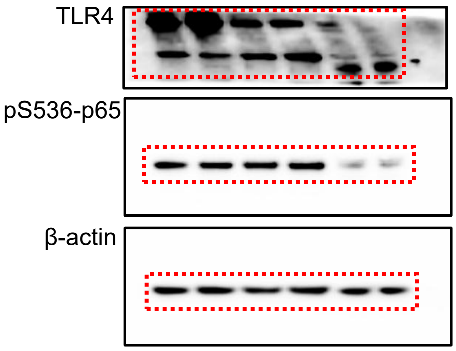

Sup. Fig.S2B

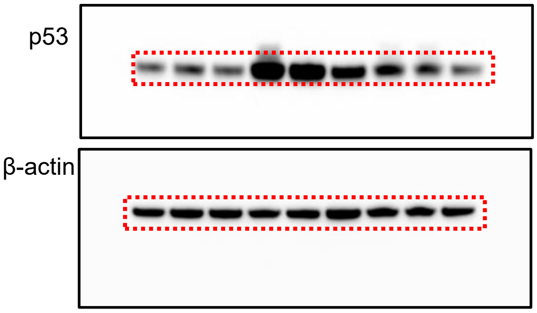

Sup. Fig.S2C

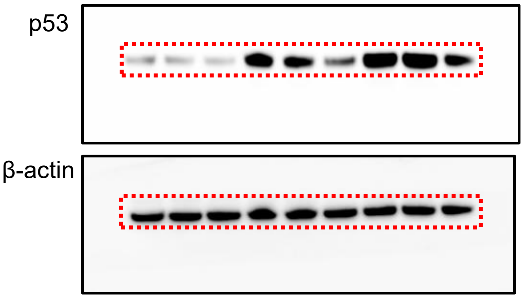

Sup. Fig.S2D

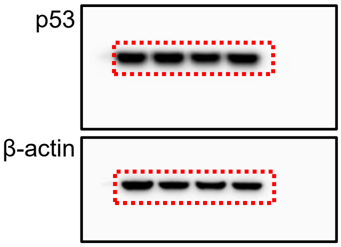

Sup. Fig.S3A

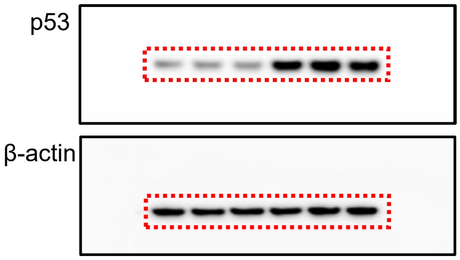

Sup. Fig.S3C

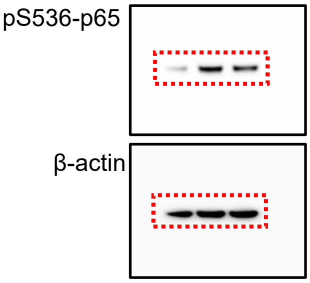

Sup. Fig.S3D

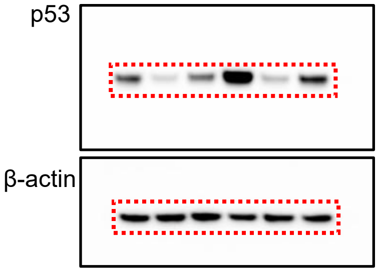

Sup. Fig.S3F

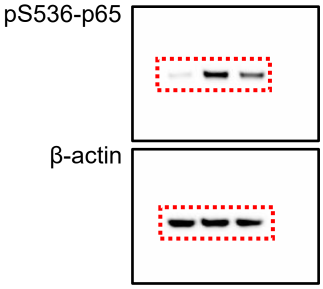

Sup. Fig.S3G

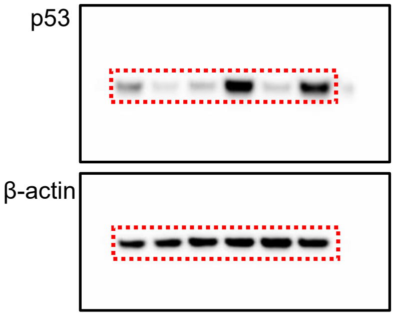

Sup. Fig.S5A

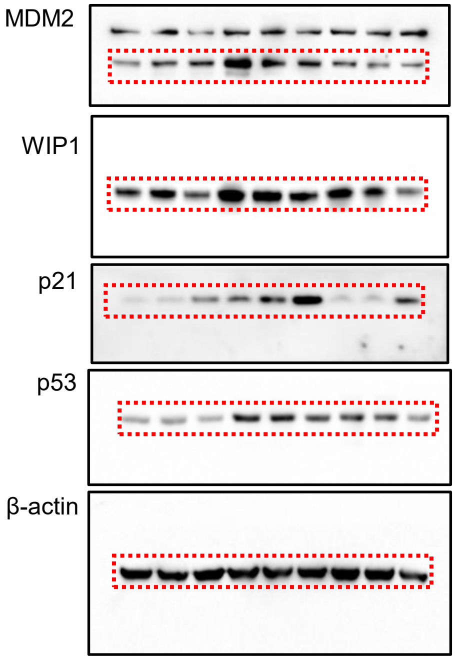

Sup. Fig.S5B

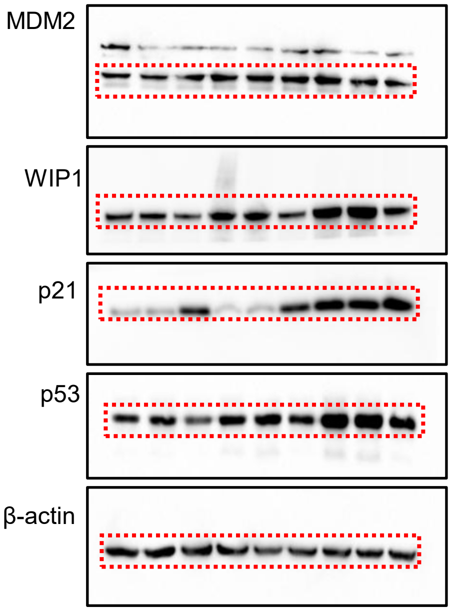

Sup. Fig.S7A

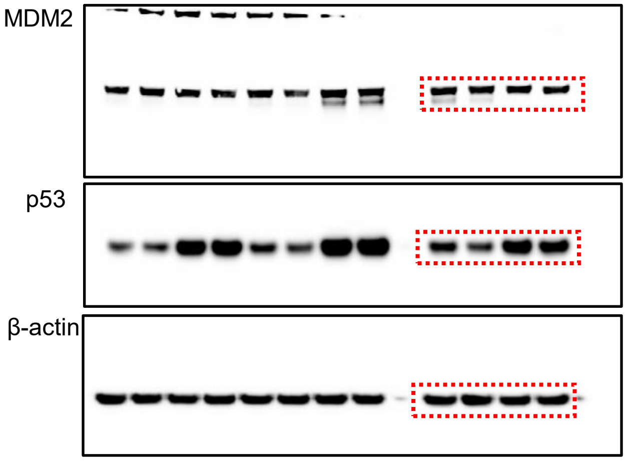

Sup. Fig.S8C

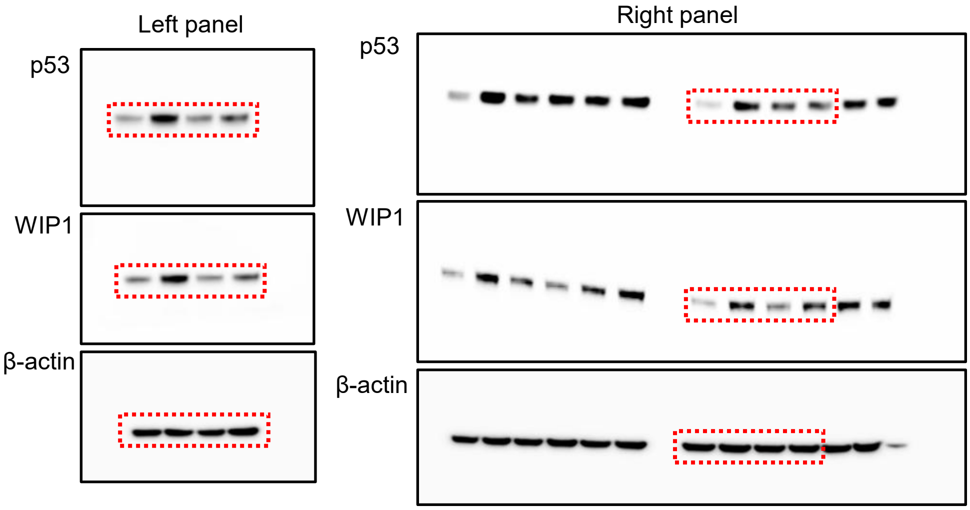

Sup. Fig.S8D

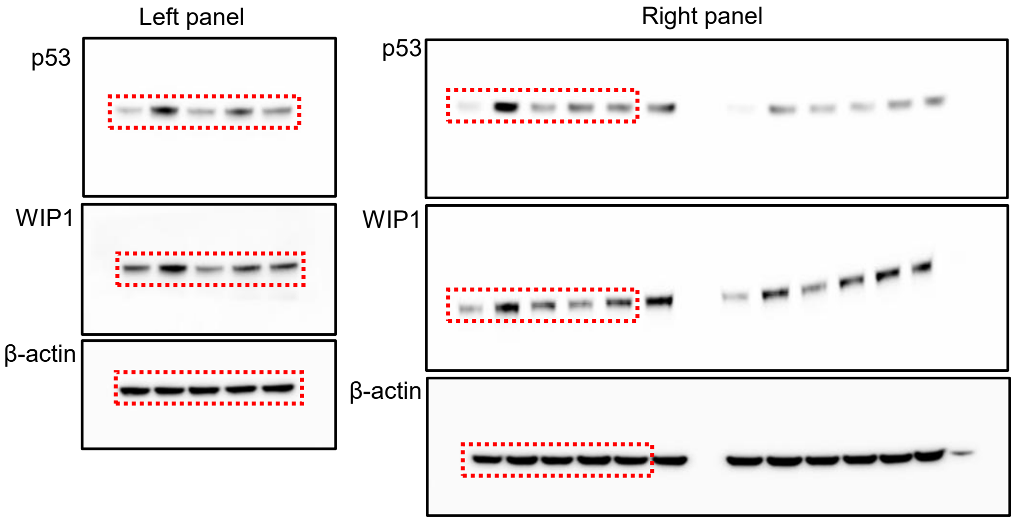

Supplement: Supplementary file 5 — Uncropped Western blots [file 41419_2026_8756_MOESM5_ESM.pdf]
